# Adrenomedullin from spinal cord injury drives heterotopic ossification in skeletal muscle via a neuro–immune–bone axis

**DOI:** 10.1038/s12276-026-01836-7

**Published:** 2026-09-07

**Authors:** Jiewen Chen, Zilong Wang, Xiaolin Zeng, Xiaoyu Wu, Gang Li, Nanyan Miao, Yilong Deng, Di Zhang, Xi Chen, Hongxi Lai, Yong Wan, Le Wang, Xiang Li

**Affiliations:** 1 Department of Spine Surgery, The First Affiliated Hospital, Sun Yat-sen University, Guangzhou, China; 2 Guangdong Province Key Laboratory of Orthopaedics and Traumatology, Guangzhou, China; 3 Department of Orthopedic Surgery, The Second Affiliated Hospital of Guangzhou University of Chinese Medicine, Guangdong Provincial Hospital of Chinese Medicine, Guangzhou, China; 4 Department of Joint Surgery, The First Affiliated Hospital, Sun Yat-sen University, Guangzhou, China; 5 Division of Spine Surgery, Department of Orthopedic Surgery, Nanjing Drum Tower Hospital, Affiliated Hospital of Medical School, Nanjing University, Nanjing, China; 6 Department of Orthopaedics, Union Hospital, Tongji Medical College, Huazhong University of Science and Technology, Wuhan, China; 7 Department of Plastic Surgery, Sun Yat-Sen Memorial Hospital, Sun Yat-Sen University, Guangzhou, China; 8 Faculty of Medicine, Macau University of Science and Technology, Taipa, China

**Keywords:** Targeted bone remodelling, Osteoimmunology

## Abstract

Neurological heterotopic ossification can rapidly arise in the periarticular muscles following spinal cord injury. However, the specific mechanism of how the injured central nervous system regulates heterotopic bone formation in peripheral tissues remains largely unexplored. Here we first demonstrate through single-cell RNA sequencing and spatial transcriptomic analysis that fibroadipogenic progenitor cells (FAPs) contribute to heterotopic ossification in injured muscle tissues following spinal cord injury. Mechanistically, we define a neuro–immune–bone axis in which endothelial tip cells within the injured spinal cord serve as a major source of adrenomedullin (ADM), which acts on muscle-resident regulatory T cells (T_reg_) through RAMP2, promoting their expansion and enhancing their pro-osteogenic phenotype characterized by increased BMP-2 and TGF-β production. These ADM-activated muscle-resident T_reg_ subsequently promote the osteogenic differentiation of FAPs. Conditional knockout of *Ramp2* on T_reg_ or inhibition of spinal cord-derived ADM suppresses osteogenic differentiation of FAPs and subsequent neurological heterotopic ossification. Collectively, this work elucidates a mechanistic paradigm in which the injured central nervous system remotely orchestrates peripheral pathology through long-range cellular mediators, redefining our understanding of cross-system communication after neurologic damage.

## Neuro–immune–bone axis drives ossification after spinal cord injury

Neurological heterotopic ossification (NHO) is a severe complication following spinal cord injury, where ectopic bone forms in soft tissues, often leading to pain and joint fusion. Current treatments only alleviate symptoms, highlighting a need for deeper understanding of NHO’s pathogenesis. This study explores the mechanisms behind NHO, focusing on the role of fibroadipogenic progenitors and muscle-resident regulatory T cells (T_reg_). Using spatial transcriptomics and single-cell RNA sequencing, the authors identified a neuro–immune–bone axis where spinal cord-derived adrenomedullin promotes T_reg_ expansion, enhancing their pro-osteogenic phenotype and driving fibroadipogenic progenitors to form bone. The study found that adrenomedullin from endothelial tip cells in the injured spinal cord acts through RAMP2 on T_reg_, linking spinal cord injury to peripheral ossification. These findings suggest potential therapeutic targets for NHO provide insights into central nervous system control over peripheral tissue homeostasis.

This summary was initially drafted using artificial intelligence, then revised and fact-checked by the author.

## Introduction

Neurological heterotopic ossification (NHO) is a severe complication following spinal cord injury (SCI), characterized by ectopic bone forms in periarticular soft tissues^1,2^. NHO often forms at the sites of peripheral injuries, commonly around the hip, knee and shoulder joints, causing severe pain and joint deformation that can progress to complete joint fusion^3,4^. Notably, the first-line drugs for heterotopic ossification (HO) can only relieve pain but cannot eliminate ossified lesions. In addition, high recurrence rates persist even following local surgical resection. This therapeutic stalemate underscores a critical gap in our understanding of the condition’s pathogenesis. This observation that NHO frequently originates at sites of concomitant peripheral injury, positions it as a local manifestation of a systemic dysregulation initiated by the SCI, revealing a fundamental misinterpretation of repair signals that erroneously directs soft tissues toward an osteogenic fate. Therefore, elucidating the mechanisms by which central injury orchestrates this aberrant peripheral ossification is not only immediately necessary for developing effective interventions for NHO but also offers key mechanistic insights into the fundamental principles of cross-system homeostasis regulated by the central nervous system (CNS).

Fibroadipogenic progenitors (FAPs) represent a key population of mesenchymal progenitor cells residing in the interstitial space of skeletal muscle^5^. Their role in muscle injury is important, as they aggregate and proliferate transiently to aid in the repair process^6,7^. Recent studies have highlighted that PDGFRα^+^ FAPs, which are present in various adult tissues, exhibit osteogenic potential both in vitro and in vivo, particularly in contexts where mutations associated with fibrodysplasia ossificans progressiva are present^8^. This suggests that FAPs are not only crucial for muscle regeneration but also play a pivotal role in abnormal bone formation. The ability of FAPs to differentiate into osteogenic lineages under certain pathological conditions underscores their dual potential in tissue regeneration; therefore, it is necessary to prevent unwanted ossification of FAPs in pathological conditions.

Recently, a specific population of tissue regulatory T cells (T_reg_) was identified in skeletal muscle that displayed a specific transcriptome, named as muscle-resident T_reg_, which control over nonimmunological processes^9–11^. T_reg_ in nonlymphoid tissues such as muscles were shown to exert key functions in the control and maintenance of tissues^9–11^. When acute injury occurred, muscle-resident T_reg_ accumulated in the repaired skeletal muscle and expressed the phenotypic markers such as Areg and ST2, which play an important role in supporting muscle integrity and repair^12^. In addition, the muscle-resident T_reg_ populations in muscle from Duchenne muscular dystrophy mouse model exhibited more pronounced clonal expansions compared to those observed in cardiotoxin (CDTX)-injured muscles^10^. Therefore, it is conceivable that the quantity or function of muscle-resident T_reg_ may be altered in the muscular-relative diseases.

Despite these insights, it remains currently unknown whether SCI modulates muscle T_reg_ and their phenotype as well as whether T_reg_ are required for the NHO. Indeed, the central nervous system (CNS) plays a pivotal role in regulating bone metabolism through the secretion of neuropeptides^13^. Following SCI, the integrity of the blood–brain barrier is compromised, resulting in increased permeability that allows neuropeptides to infiltrate surrounding muscle tissues^14^. Among the neuropeptides that are upregulated post injury, adrenomedullin (ADM) has garnered attention due to its dual role in bone metabolism and immune modulation^15^. ADM is expressed at high levels in osteoblasts and chondrocytes in mouse and rat embryos^13^. In the collagen-induced arthritis model, ADM induced the emergence of T_reg_ cells and prevented the systemic bone loss by maintaining osteoblastic activity^16^. Even less is known about the generation and function of ADM in injured spinal cord and HO. In particular, their regulation on T_reg_ is yet to be addressed.

In this study, we establish the NHO model and firstly used spatial transcriptomics, which combines single-cell RNA sequencing (scRNA-seq) with tissue section imaging. This approach allows us to obtain gene expression information and the spatial context of individual cells from muscle tissue sections from NHO area, as well as to identify cell–cell interactions and their responses to the ossification environment. Critically, we uncover a neuro–immune–bone axis orchestrating NHO pathogenesis. In this axis, FAPs serve as the primary osteogenic effector cells, whereas muscle-resident T_reg_ function as immune intermediates that link SCI to FAP osteogenic conversion. T_reg_ depletion demonstrates the requirement of T_reg_ for NHO formation, and NHO-associated muscle-resident T_reg_ exhibit increased production of osteogenic factors, including BMP-2 and TGF-β. Mechanistically, we identify endothelial tip cells within the injured spinal cord as a major source of ADM. Spinal cord-derived ADM acts on muscle-resident T_reg_ through RAMP2, promoting their expansion and enhancing their pro-osteogenic phenotype, thereby inducing FAP osteogenic differentiation and subsequent NHO formation. Our findings not only identify potential novel therapeutic targets for NHO but also provide a mechanistic understanding of how a damaged CNS orchestrates peripheral pathology through long-range cellular and molecular signals.

## Materials and methods

### Bulk RNA-seq and data analysis

Total RNA was obtained from C57BL/6 mice (*n* = 6, female, 3–5 weeks, 18–20 g, specific pathogen-free grade) of CDTX (*n* = 3) and NHO group (*n* = 3). According to the manufacturer’s instructions, total RNA was extracted using a TRIzol reagent kit (Invitrogen) and quantified using NanoDrop ND-1000 (NanoDrop). The RNA integrity was determined by the Bioanalyzer 2100 (Agilent) with a RNA integrity number value >7.0 and verified by electrophoresis using denaturing agarose gel. poly(A) RNA is purified from 1 μg total RNA using Dynabeads Oligo (dT) 25-61005 (Thermo Fisher) using two rounds of purification. Then, under 94 °C for 5–7 min, the poly(A) RNA was broken up into small pieces using Magnesium RNA Fragmentation Module (NEB, cat. no. e6150). The cleaved RNA fragments were reverse-transcribed to create the complementary DNA by SuperScrip II Reverse Transcriptase (Invitrogen, cat. no. 1896649). Single- or dual-index adapters were ligated to the fragments, and size selection was performed with AMPureXP beads. After the heat-labile UDG enzyme (NEB, cat. no. m0280) treatment of the U-labeled second-stranded DNAs, the ligated products are amplified with PCR under the following conditions: initial denaturation at 95 °C for 3 min; eight cycles of denaturation at 98 °C for 15 s, annealing at 60 °C for 15 s and extension at 72 °C for 30 s and, finally, final extension at 72 °C for 5 min. The resultant cDNA library’s average insert size was 300 ± 50 bp. At last, we performed the 2× 150 bp paired-end sequencing (PE150) on an illumina Novaseq 6000 (LC-Bio Technology) following the vendor’s suggested procedure.

Quantize normalization and subsequent data processing were performed using Agilent GeneSpring GX software, version 11.5.1. Volcano plot and heat map were analyzed using an online bioinformatics tool (www.bioinformatics.com.cn) for data analysis and visualization. Normalized data are accessible on NCBI database with the assigned GEO accession number GSE245470.

### SCI scRNA-seq

The scRNA-seq data pertaining to SCI was downloaded from the GEO database (GSE162610) and subsequently clustered by the Seurat package (version 4.1.0). Highly variable genes were calculated using Seurat ‘Find Variable Genes’ function. Using HVGs, principal component analysis (PCA) was conducted, and the top 15 principal components were chosen for uniform manifold approximation and projection (UMAP) dimensionality reduction. ‘Harmony’ was applied to remove batch effects, and UMAP visualized 66,178 single cells. Unbiased clustering generated 13 clusters annotated to 13 known cell types based on canonical marker genes. Differentially expressed genes (DEGs) (adjusted *p* < 0.05, |log_2_FC| > 0.5) were identified with Seurat’s ‘FindMarkers’ function.

To perform further cluster analysis of specific cell type, we extracted cell clusters in the integrated Seurat object using the subset function and repeated the above cluster analysis and DEG analysis steps for specific cell type. The gene sets of Kyoto Encyclopedia of Genes and Genomes (KEGG) and Gene Ontology (GO) pathways were obtained from MSigDB (https://www.gsea-msigdb.org/gsea/index.jsp). GO and KEGG pathways analysis of cell subset was performed using ClusterProfiler (version 4.2.2).

### NHO scRNA-seq

#### Tissue dissociation

Single-cell isolation hind-limb muscles were dissected from mice in each experimental condition (three each to minimize individual variability). Skeletal muscle single-cell suspension was prepared by collagenase/dispase digestion. In brief, each tissue was minced in 2.5 ml digestion solution (1 U/ml collagenase B and 1 U/ml dispase II (Thermo Fisher) in phosphate-buffered saline (PBS)) and incubated at 37 °C for 1 h. The reaction was terminated by adding 10 ml PBS containing 10% FBS. The mixture was then fltered through a 40-µm cell strainer. A Dead Cell Removal Kit (Miltenyi Biotec) was used to remove cells with low viability. The live cells were resuspended in PBS containing 0.04% bovine serum albumin. Subsequently, the live cells were centrifuged at 250*g* for 5 min at 4 °C to obtain a cell pellet. The cell pellet was resuspended and washed twice. The cells were diluted to a final concentration of 1 × 10^6^/ml in PBS with 0.04% bovine serum albumin. All the above steps were completed under aseptic conditions and were performed on ice to preserve cell viability.

#### ScRNA-seq and data processing

The volume of single-cell suspension that was required to generate 10,000 single-cell GEMs (gel beads in the emulsion) per sample was loaded onto the 10× Chromium single-cell platform (10x Genomics). Libraries were prepared using the Chromium v3 Single Cell 3′ Library and Gel Bead Kit v3 (10x Genomics) according to the manufacturer’s specifications. Generation of GEMs, barcoding, GEM reverse transcription, complementary DNA amplification and library construction were all performed according to the steps of the instructions. Final library quantification were performed using Qubit, followed by sequencing on Illumina NovaSeq 6000 in double-ended 150-bp mode. Raw sequence data were aligned to the reference genome of the Ensembl database (https://asia.ensembl.org/index.html), and cell numbers along with unique molecular identifiers (UMIs) were estimated, using the Cell Ranger (version 7.0.0), the single-cell software suite from 10x Genomics. Downstream analyses were performed using the Seurat R package (version 4.3.0). To remove cells with poor quality, cells with high fraction of mitochondrial transcripts, with low UMI counts and with low number of detected genes were removed for analysis. Data were normalized using NormalizeData function, and then logtransformed for the subsequent analyses.

#### ScRNA-seq data analysis

The top 2,000 variable genes were identified using the FindVariableFeatures function. The data were scaled according to the mitochondrial percentage using the ScaleData function. Different samples were integrated using the ‘anchors identify’ step and the Harmony function to correct batch effects. PCA analysis was run, using variable genes, for the top 20 components. Unsupervised clustering was performed with the functions FindNeighbors and FindClusters, and clusters were then visualized with UMAP. Differential gene expression analysis among clusters was performed using the Seurat FindAllMarkers or FindMarkers function, with the active assay of the Seurat object set as ‘RNA’. Genes with adjusted *P* values <0.05 were selected as DEGs. For each cell cluster, we assigned the cell-type labels using statistical enrichment for sets of marker genes and manual evaluation of gene expression for small sets of known marker genes.

#### Cell–cell interaction analysis

Using the R package CellChat (version 2.1.1), cell–cell communications were predicted and quantified. Specifically, we isolated the cell populations to be studied from the scRNA-seq datasets within each of the two experimental conditions, that is, Control Sham and experimental NHO. Using CellChat’s normalizeData function, each gene expression matrix underwent library-size normalization, log-transformation with a pseudocount of 1. We then loaded a gene expression matrix and its associated cell meta data to create a CellChat object for each of the two experimental conditions using the createCellChat function. We set the ligand–receptor interaction database as CellChatDB.mouse and filtered genes in each CellChat object by retaining the signaling genes to reduce computational load. Overexpressed genes and overexpressed ligand–receptor interactions were identified using the identify overexpressed genes and identify overexpressed interactions functions. Then, we calculated the cell–cell communication probability by the computeCommunProb function. The cell–cell communications among different cell types within each experimental condition were visualized using the circle plot. Finally, we compared the number of interactions/interaction strength between the two groups.

#### GO and pathway enrichment analysis

The gene sets of GO pathways were obtained from MSigDB (https://www.gsea-msigdb.org/gsea/index.jsp). GO enrichment analysis was performed with the R package ClusterProfiler (version 4.2.2).

#### Analysis of specific cell types

To perform further cluster analysis of T cell, we extracted cell clusters in the integrated Seurat object using the subset function and repeated the above cluster analysis and DEG analysis steps for T cell.

### Spatial transcriptomic

#### Tissue imaging, library preparation and sequencing

Tissue samples were embedded with OCT (TissueTek, Sakura). Samples were prepared for cryo-sectioning at −22 °C and cut at 10 μm using a Leica CM3050 S cryostat. Brightfield images were captured at 20× resolution using a Leica Aperio VERSA whole-slide scanner. The sections were placed on chilled Visium Tissue Optimization Slides (3000394, 10x Genomics) and Visium Spatial Gene Expression Slides (2000233, 10x Genomics) and adhered by warming the back of the slide. Following the manufacturer’s instructions, sequencing libraries were created using the Visium Spatial Gene Expression Slide and Reagent kit (1000187, 10x Genomics). In a nutshell, a frozen tissue segment (10 μm) was placed on a gene expression slide’s capture region (6.5 mm × 6.5 mm with 5,000 barcoded dots) and then stained with hematoxylin and eosin (H&E). Brightfield images were taken. The tissue was permeabilized for 24 min, which the tissue optimization time-course trials determined to be the ideal moment. In the presence of actinomycin, cDNA was synthesized overnight using SuperScript III Reverse Transcriptase. Libraries were loaded at 300 pM and sequenced on a NovaSeq 6000 System (Illumina) using a NovaSeq S4 Reagent Kit (Illumina). Sequencing was performed using the following read protocol: read 1, 28 cycles; i7 index, 10 cycles; i5 index, 10 cycles; read 2, 90 cycles.

#### Spatial transcriptomics analysis

The 10x Genomics official tool kit Space Ranger (version 3.0.0) was used to perform sequencing read alignment, fiducial/tissue detection and spot barcode/UMI counting on the spatial transcriptomic data independently for each slice utilizing an H&E-stained brightfield image and fastq files as inputs. Reads were mapped to the Mouse Genome (mm10), and Seurat was used to import the gene-spot matrix for further analysis and visualization. Only spots that have been identified as covering tissue were kept. Seurat sctransform was used to standardize the combined data. The ‘RunPCA’ function was used to accomplish linear dimensional reduction with PCA. In accordance with the top 30 PCA components, a shared nearest neighbor (SNN) graph was created using the ‘FindNeighbors’ function. The Louvain algorithm (resolution of 0.4) was able to cluster the spots using the SNN graph and the ‘FindClusters’ function. The ‘RunUMAP’ function was used to execute UMAP dimensional reduction and view the spots in a two-dimensional space. The molecular signature of each nucleus/spot cluster was obtained by comparing the transcriptome of each cluster with that of the other clusters using a likelihoodratio test (test.use: ‘bimod’) implemented in the ‘FindMarkers’ function of Seurat. The significance threshold was set to an adjusted *P* value <0.05 and a log_2_ fold change >0.25.

Integration of the spatial transcriptomic data with the single nucleus RNA sequencing (snRNA-seq) data: to integrate the spatial transcriptomic data with the snRNA-seq data and predict the underlying cellular composition for each spot that contained multiple nuclei, we applied the label transfer workflow of Seurat to assign each spot prediction score for the subpopulations obtained from the snRNA-seq data analysis. Following the label transfer, the spot-level cellular composition of the spatial transcriptomic data was visualized with the st.pl.deconvolution_plot function of stLearn (version 0.3.1). Noise labels were filtered for better visualization based on quantile (threshold of 0.5).

Signature scoring of relevant gene derived from spatial transcriptomics signatures was performed with the AddModuleScore function with default parameters in Seurat. Spatial feature expression plots were generated with the SpatialFeaturePlot function in Seurat (versions 4.3.0).

The expression activity of a given gene set/pathway in each nucleus/ST spot was quantified by calculating the gene set/pathway activity score for each nucleus or spot using the method implemented in Single Cell Signature Explorer. The mean of the computed scores across the nuclei/spots in each sample served as a proxy for the expression activity of a pathway. Spatial feature expression plots were generated with the SpatialFeaturePlot function in Seurat (versions 4.3.0). Based on the calculated gene set score of each spatial transcriptomic spot, the given gene set of the spots in the NHO tissue sections of the cellular regions of the FAPs were detected for differences using the Wilcoxon rank-sum test in the ‘FindMarkers’ function of Seurat at default settings. The significance threshold was set to a Bonferroni-adjusted *P* value <0.05 and an absolute of the log_2_ fold change >0.25.

### FAPs cell culture

FAPs obtained from flow sorting of hamstring muscle samples, cells were seeded at 3,000 per square centimeter in α-MEM supplemented with 20% FBS, 1% antibiotics and 10 ng/ml β-FGF (R&D Systems), in a humidified incubator at 37 °C and 5% CO_2_. When confluent, the cells were detached using a trypsin (0.25%)-EDTA (1 mM; Thermo Fisher Scientific) solution and subcultured in 6- or 12-well plates. Cellular immunofluorescence (IF) staining was performed for the identification of FAPs.

### T_reg_ culture

Spleen T_reg_ and muscle-resident T_reg_ were isolated from mice as indicated. In brief, spleens were mechanically dissociated into single-cell suspensions, and red blood cells were removed using red blood cell lysis buffer. Injured hindlimb muscle tissues were minced and enzymatically digested as described above to obtain single-cell suspensions. T_reg_ were enriched and sorted from spleen or muscle single-cell suspensions by flow cytometry. Isolated T_reg_ were cultured in RPMI 1640 medium supplemented with 10% FBS, 1% antibiotics, anti-CD3/CD28 stimulation and recombinant IL-2.

### CM assay

The culture supernatants of T_reg_ were collected, centrifuged at 500*g* for 5 min to remove cellular debris, and used as T_reg_-conditioned medium (T_reg_-CM). For the T_reg_-CM assay, FAPs were seeded in osteogenic medium and treated with T_reg_-CM at a 1:10 ratio. The medium was changed every 3–4 days with freshly prepared osteogenic medium containing the corresponding T_reg_-CM. After 14 days of osteogenic induction, FAP mineralization was assessed by Alizarin Red S staining and quantified as described above.

### hUVECs cell culture and generation of endothelial Tip cells

Human primary cell lines, human umbilical vein endothelial cells (hUVECs), were purchased from Zhong Qiao Xin Zhou Biotechnology and cultured in endothelial cell medium (no. ZQ-1304; Zhong Qiao Xin Zhou Biotechnology), within a humidified chamber at 37 °C with 5% CO_2_. When cultured to passage 2 or 3, they were frozen in liquid nitrogen. Only hUVECs cultured up to passage 6 were used for experiments.

Use 2% w/v agarose as a mold to form cell spheres while preventing cells from adhering to the surface of the mold. Heat to melt and pour into three-dimensional Petri dishes, solidify and place in six-well plates to culture the cells. The cells were inoculated with 200 μl of cell suspension, medium was added after 15 min and the cells were aggregated to form cell spheres for 24 h. The cell spheres were then seeded into 2.5% w/v methacryloylated gelatin, which was light-cured, then incubated in special medium with 25 ng/ml of VEGF for 24 h. The cells were induced to generate tip cells according to our previous publication^17^, which were then used for further experiments.

### mRNA extraction and RT–qPCR analysis

Following the manufacturer’s instructions, total RNA was extracted from the cells or homogenates of tissue using a TRIzol reagent kit (Invitrogen), and 2 µg of total DNA-free RNA was used to create cDNA using the ReverTra Ace qPCR RT Kit (Toyobo, Osaka, Japan). In 96-well plates, the reactions were set up using 1 µl of cDNA and Thunderbird SYBR qPCR Mix (Toyobo, Osaka, Japan), to which forward and reverse PCR primers specific to the desired gene were added. Reverse-transcription quantitative PCR (RT–qPCR) was performed under the following conditions: 95 °C for 5 min, followed by 40 cycles of 95 °C for 10 s and 55 °C for 34 s.

These analyses were performed to detect *Adm*, *Pecam1*, *Apln*, *Trp53i11*, *Runx2*, and Bglap expression. Gapdh was used as an internal control. The primer sequences are shown in Supplementary Table 1.

### Protein extraction and western blot analysis

Total protein was extracted, and the protein concentration was determined by BCA assay. A 10% SDS–polyacrylamide gel electrophoresis (Asegene) was loaded with 20 µg of total protein, and the separated proteins were transferred by electro blotting to PVDF membranes. The membranes were blocked with 5% nonfat dry milk in TBST (50 mM Tris, pH 7.6, 150 mM NaCl, 0.1% Tween 20) and incubated with the primary antibody overnight at 4 °C in 5% nonfat dry milk in TBST. Primary antibody information is presented in Supplementary Table 2. Blots were washed three times with TBST for 5 min each time, and membranes were incubated with horseradish peroxidase (HRP)-conjugated secondary antibodies for 1 h at room temperature and washed three times with TBST. Immunolabeling was detected using ECL reagent (Thermo Fisher Scientific). Chemiluminescence was detected using an enhanced chemiluminescence system.

### ELISA

Enzyme-linked immunosorbent assay (ELISA) was performed to quantify ADM, UCN2, BMP-2 and TGF-β levels in serum samples or cell culture supernatants. Blood samples were centrifuged at 1,800*g* for 10 min at 4 °C and serum fractions were stored at −80 °C. The protein levels of ADM in sera were determined using an ELISA kit according to the recommendations of the manufacturer (HuaMei). For cell culture experiments, culture supernatants were collected from endothelial tip cells or isolated mouse T_reg_ after the indicated treatments. The supernatants were centrifuged at 500*g* for 5 min to remove cellular debris and were then subjected to ELISA analysis. ADM levels in endothelial tip cell-conditioned medium were measured using an ADM ELISA kit. BMP-2 and TGF-β levels in T_reg_-conditioned medium were measured using mouse BMP-2 and TGF-β ELISA kits, respectively. Absorbance was measured using a Multiskan Sunrise microplate reader (TECAN). Concentrations were calculated according to the standard curves generated for each ELISA kit. Detailed information on the ELISA kits is provided in Supplementary Table 2.

### Flow cytometric analysis and sorting

Muscle samples were processed for flow cytometry as stated below. Single cells from muscles were separated using a skeletal muscle dissociation kit (Miltenyi Biotech, Bergisch Gladbach) and a GentleMACS Dissociator tissue homogenizer (Miltenyi Biotec). Single-cell suspensions were prepared and were stained with fluorochrome-conjugated antibodies. Data were collected on a BD LSRII flow cytometer (BD Biosciences) and analyzed with FlowJo v. 10.7.1 software (Tree Star). Data were acquired as the fraction of labeled cells within a live-cell gate set for 50,000 events. For flow cytometric sorting, cells were stained with specific antibodies and isolated on a BD FACSAria cell sorter (BD Bioscience).

### Micro-CT analyses

HO specimens were obtained from mice postmortem and fixed with 4% paraformaldehyde. For micro-computed tomography (micro-CT scanning), specimens were fitted in a cylindrical sample holder and scanned using a Scanco lCT40 scanner set to 55 kVp and 70 lA. Specimens were scanned with a mean 20-μm slice thickness. For visualization, the segmented data were imported and reconstructed as three-dimensional images using MicroCT Ray V3.0 software (Scanco Medical). HO formation was evaluated using reconstructed three-dimensional images and ectopic bone volume was calculated. To calculate HO volumes, the region of interest was drawn around the muscles containing HO and was carefully checked from three dimensions. After defining the region of interest, the HO region was defined by setting the threshold Hounsfield units (HU) to 450 HU.

### Quantification of clinical HO volume

HO volume in patients was quantified from preoperative CT images using three-dimensional segmentation. In brief, DICOM images were imported into Mimics Research 21.0 software. The region containing HO was manually identified on serial CT slices. Based on previous CT-based HO volumetric studies, mineralized HO lesions were initially segmented using a lower density threshold of 150 HU, followed by manual anatomical correction to exclude adjacent native skeletal bone, vascular calcification and imaging artifacts. The segmented HO volume was automatically calculated based on voxel volume and expressed as cubic centimeters. For patients with multiple HO lesions within the scanned region, total HO volume was calculated as the sum of all segmented lesions.

### In vitro osteogenic differentiation assay and mineralization quantification

For detecting the mineralization, FAPs were plated at a density of 2 × 10^5^ cells per well in 12-well tissue-culture plates in α-MEM supplemented with 10% FBS and 1% antibiotics. Once the cells adhered to the wells, the medium was replaced by α-MEM supplemented with 10% FBS, 1% antibiotics and 50 μg/ml L-ascorbate acid, 0.1 μM dexamethasone and 10 μM β-glycerophosphate (Sigma-Aldrich) to induce osteogenic differentiation. Cells were additionally stimulated by different conditions. The cells were cultured for 14 days at 37 °C in a 5% CO_2_ atmosphere, and the medium was changed every 3–4 days.

Cells were washed three times with PBS and fixed with 70% ethanol for 10 min. After three distilled-water rinses, the cells were stained with a 40 mM alizarin red S (Sigma-Aldrich) solution (pH 4.1) for 10 min to visualize matrix calcium deposition. The remaining dye was washed three times with distilled water, and photographs were taken of the stained cells. For quantification, the calcium deposits were distained with 10% cetylpyridinium chloride in 10 mM sodium phosphate (pH 7.0), then the extracted stain was transferred to a 96-well plate, and the absorbance of the samples was measured at 570 nm using a microplate reader (Tecan)^18,19^.

### In vivo treatment with recombinant mouse ADM or anti-CD25 neutralizing antibody

Recombinant ADM was purchased from SinoBiological. C57BL/6 mice were injected intrathecal injection with recombinant ADM at a dose of 10 nM, once daily from day 0 to day 14 post surgery.

C57BL/6 mice were also injected intraperitoneally with 200 μg daily of anti-CD25 neutralizing antibody (Absolute antibody, cat. no. Ab01107, clone PC-61.5.3) or 200 μg control IgG monoclonal antibody (Bio X Cell, cat. no. BE0091, clone PIP) from day 0 to day 14 post surgery.

### ADM 22-52 (ADM receptor antagonist, AMA) pharmaceutical inhibition

In the FAP-T_reg_ co-culture system, for ADM treatment, the cells were treated with 10 nM ADM for 30 min. For ADM receptors antagonizing, 1 μM ADM receptor antagonist (AMA) was added into the medium for 30 min before the ADM pretreatment, the control group was added an equal volume of PBS. In NHO mouse model, the mice were randomly assigned to different treatment groups and NHO + AMA group was treated with AMA (hind-limb intramuscular injection 50 µg per day) from day 0 to day 14 post surgery. Control group was injected intraperitoneally with an equal volume of PBS.

### Histological analysis

Tissue samples were first fixed for 24 h in 4% paraformaldehyde, then decalcified for 4 days in 14% EDTA. Every 24 h, the solution was replaced with 10%, 20% and 30% sucrose/PBS. Tissues were mounted in optimum cutting temperature compound, frozen in isopentane in liquid nitrogen and stored at −80 °C. Using a Leica Cryostat CM1950, tissue samples were sectioned at a thickness of 8 µm. To evaluate general structures and visualize HO formation within the tissues, H&E as well as Von Kossa (VK) and Safranin O-Fast Green (SOFG) staining were performed.

### IF staining

The tissue sections and cultured cells were fixed in 4% paraformaldehyde for 30 min and permeabilized with 0.3% Triton X-100 for 30 min. After three times wash with PBS, blocking was performed with 5% normal donkey serum for 1 h. The tissue sections and the cells were incubated overnight at 4 °C with primary antibodies. Detailed antibody information is presented in Supplementary Table 2. After washing three times in PBS, the primary antibodies were probed with Alexa Fluor 594 donkey anti-rabbit, Alexa Fluor 488 donkey anti-mouse or Alexa Fluor 488 donkey anti-rat secondary antibodies for 1 h at room temperature. Finally, the coverslips were washed in PBS three times and mounted using Prolong Gold Antifade Reagent containing 4′-6-diamidino-2-phenylindole (DAPI) (Molecular Probes, Invitrogen). All images were observed using an Olympus BX63 microscope (Olympus, Japan).

### *Adm* knockdown

For local knockdown of *Adm* in vivo, recombinant adeno-associated virus (AAV) serotype 9 vector expressing a short hairpin RNA (shRNA) directed at *Adm* (AAV-sh*Adm*) (GeneChem) or a control hairpin (AAV-shCtrl) was used. siRNA sequence is presented in Supplementary Table 3. The shRNA expression was driven by a mouse U6 promoter (pol III) and used GFP as a reporter. The final virus in PBS had a titer of 4.0 × 10^12^ viral particles per milliliter. AAV carrying GFP (AAV–GFP) was simultaneously prepared as a control vector. A total of 6 μl of AAV was infuse into the transverse section of the mouse spinal cord at a speed of 0.2 μl/min.

### Collection of clinical human samples

The Medical Ethics Committee of the First Affiliated Hospital of Sun Yat-sen University approved the procedures performed in this study. All muscle sample and blood samples were obtained with the informed consent of the patients. Muscle samples in the NHO group were collected from surgical wastes following NHO resection in patients with SCI combined with traumatic fractures. Muscle samples from the THO group were collected from surgical wastes after HO resection in patients with traumatic fractures only. Muscles of patients with only traumatic fracture, CNS injury combined with traumatic fracture but none of which had developed HO were taken as controls. And before the operation, X-rays and computed tomography scans were performed on the fracture sites of these patients. Peripheral blood serum was also collected from the above patients at the same time. The muscle samples were further stained for IF staining, HE and SOFG. Peripheral blood serum was used for further analysis of ADM levels.

#### Generation of T_reg_-specific Ramp2-knockout mice

*Ramp2*^fl/fl^ mice and *Foxp3*^Cre^ mice on a C57BL/6 background were purchased from GemPharmatech. To generate mice with T_reg_-specific deletion of *Ramp2*, *Ramp2*^fl/fl^ mice were crossed with *Foxp3*^Cre^ mice to obtain *Foxp3*^Cre^*Ramp2*^fl/fl^ mice. *Ramp2*^fl/fl^ littermates lacking the *Foxp3*^Cre^ allele were used as controls, unless otherwise indicated. *Ramp2*^fl/fl^ control and *Foxp3*^Cre^*Ramp2*^fl/fl^ mice aged 5–8 weeks were used for the NHO experiments. Mice were randomly assigned to experimental groups whenever possible, and investigators were blinded to group allocation during micro-CT quantification and histological analysis.

### Establishment of NHO model

The Medical Ethics Committee of the First Affiliated Hospital of Sun Yat-sen University approved the procedures performed in this study. The 5–8-week-old female mice were used to establish NHO animal model. The NHO mouse model was used as previously mentioned by performing a spinal cord transection between T11 and T13 together with intramuscular injection of CDTX purified from the venom of Naja pallida (Latoxan) at 0.32 mg/kg in the hamstring muscles under general anesthesia (100 mg/kg ketamine, 10 mg/kg xylazine and 1% isofluorane). Control mice underwent sham-surgery together with intramuscular injection of equal volume of CDTX or spinal cord transection together with intramuscular injection of equal volume of PBS. The mice were housed for 12 h of light–dark cycle under standard temperature conditions, with feeding and drinking water. After surgery, the bladder was emptied twice a day until bladder function was restored. In this model, NHO developed in the CDTX-injected muscle of SCI mice within 7–14 days.

For in vivo analysis of increased ADM effects on HO formation, the mice were randomly divided into three groups: the sham-surgery + CDTX, SCI + CDTX (NHO) and intramuscular injection recombinant ADM + CDTX groups. For the study of ADM reduction effects on NHO formation, the mice were randomly divided into another three groups: the SCI + CDTX (NHO), NHO + AAV-shCtrl and NHO + AAV-shADM groups. To study the influence of T_reg_ on the formation of NHO, we use CD25 neutralizing antibodies to deplete T_reg_. The mice were randomly divided into two groups: the NHO control and NHO + anti-CD25 groups. To screen for possible drugs for the treatment of NHO formation, we randomized NHO mice into two groups, one with AMA inhibitor and the other with an equal amount of PBS as a control.

At the specified point in time, all mice were sacrificed and their peripheral blood serum and limbs were collected for further experiments. Peripheral blood serum was used for further analysis of ADM levels by ELISA. The limbs could be further subjected to micro-CT and HO Volume Quantification, H&E staining, special staining, IF analysis, sequencing, flow cytometry, mRNA extraction and RT–PCR analysis, protein extraction, western blot and so on.

### Statistical analysis

**Fig. 1:**
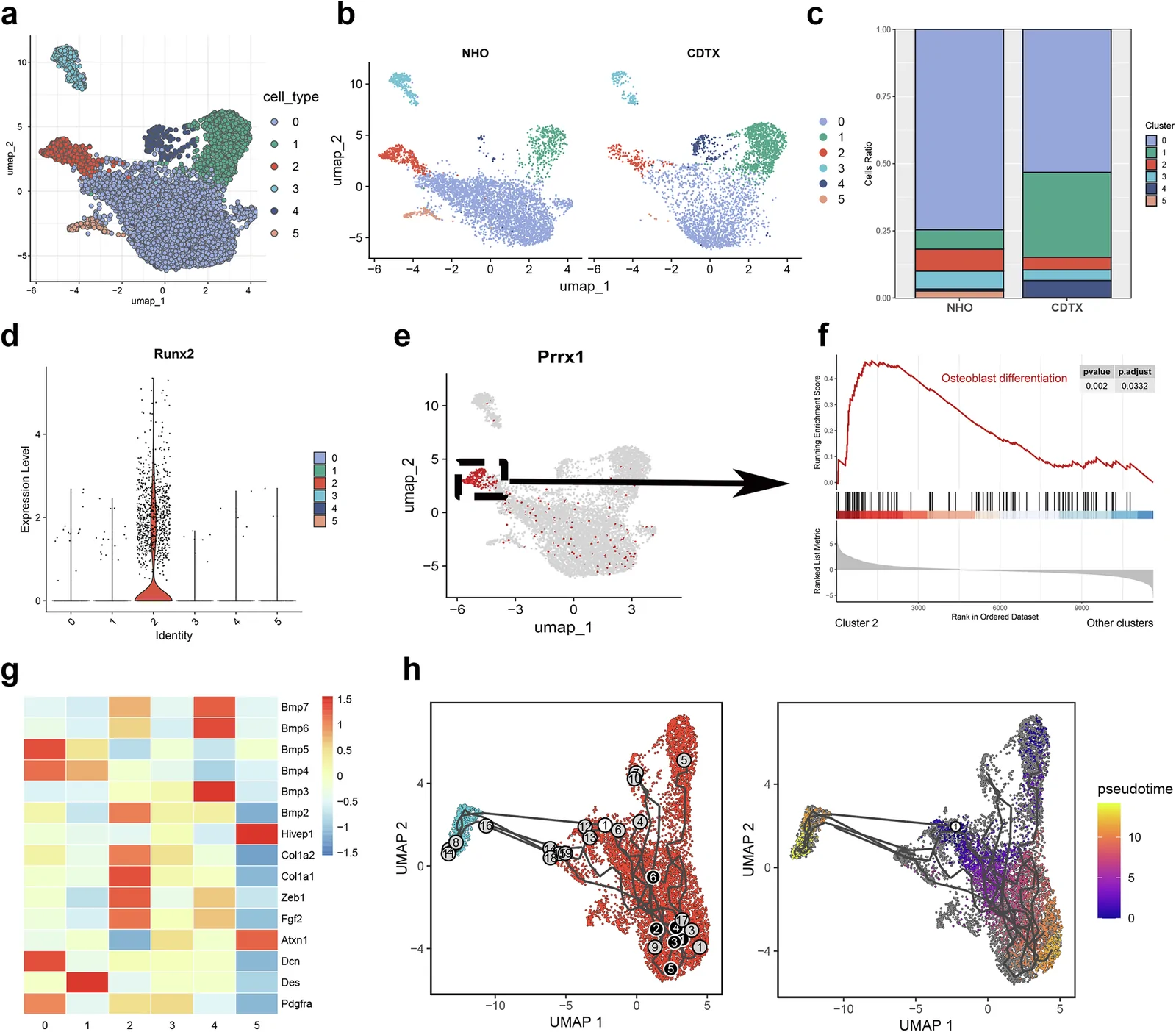
The subclusters and osteoblast differentiation of FAPs. **a** The UMAP plot shows six FAP subclusters in all samples (*n* = 6). **b** The UMAP plot shows six FAP subclusters in NHO (*n* = 3) and CDTX groups (*n* = 3). **c** The stacked bar chart shows the proportion of each FAP cluster in NHO and CDTX groups. **d** The violin plot shows osteogenic marker Runx2 was highly expressed in subcluster 2. **e**,**f** The UMAP plot (**e**) and gene set enrichment analysis (GSEA) (**f**) show a distinct subset of cells that was characterized by co-expression of Prrx1 and involved in osteoblast differentiation. **g** The heat map shows the expression level of mesenchymal and osteoblastic marker genes in six subclusters of FAP. **h** Pseudotime trajectory of FAPs and osteoblasts derived from NHO samples (*n* = 3).

**Fig. 2:**
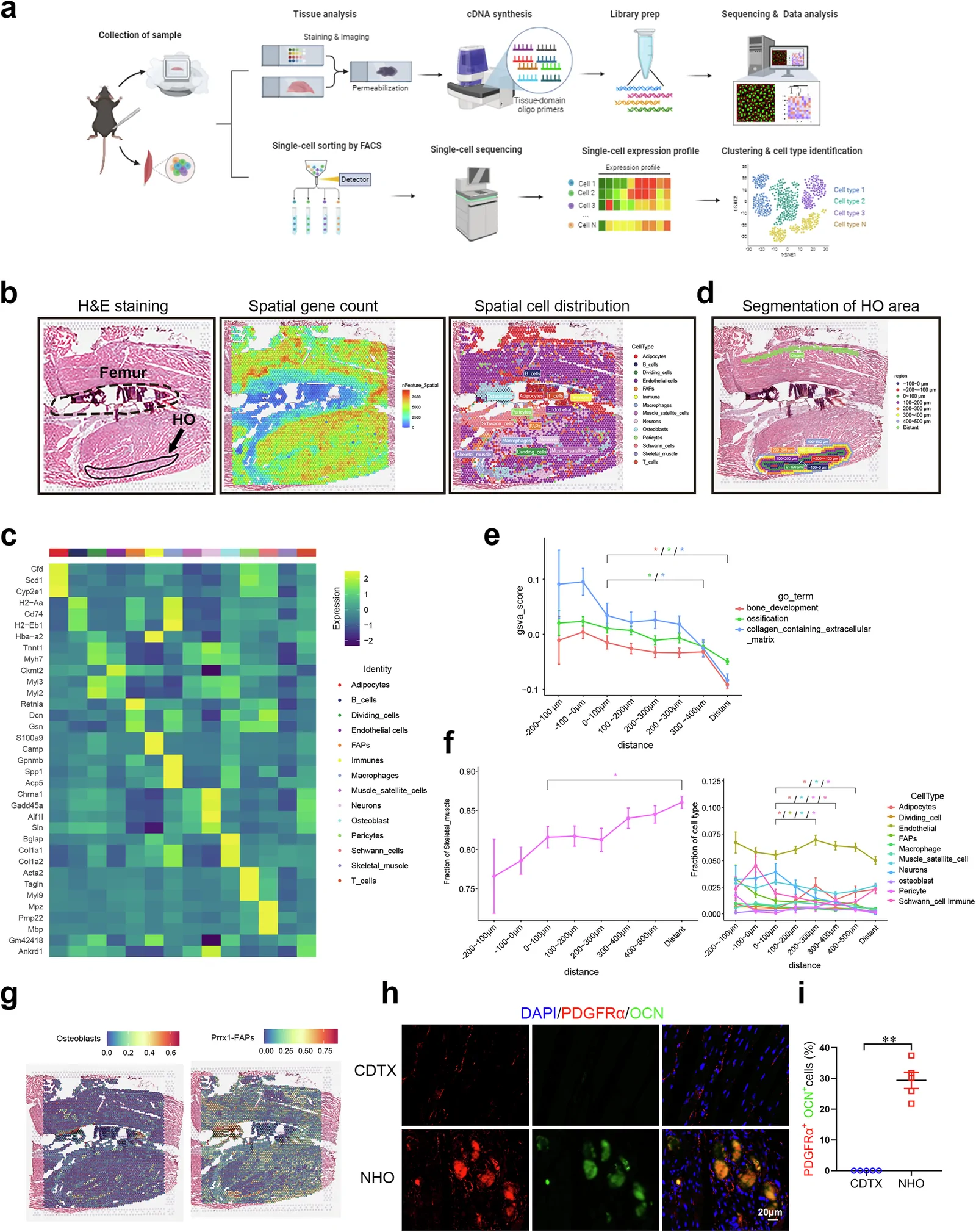
The characteristics of cell composition and spatial distribution of NHO tissue. **a** The spatial transcriptomics and scRNA-seq acquisition workflow for NHO mice. **b** The H&E staining, gene count maps and cell type maps of NHO sample. The dashed box outlines the femoral region, and the black arrow indicates the HO area. **c** The heat map shows the expression levels of marker genes for 14 cell types in annotated spatial spots. **d** The segmentation of NHO area border with each layer representing a 100-µm-wide zone. **e** The line chart shows the GSVA-score of three ossification-related pathways of different layers of NHO area. **f** The line chart shows the fraction of skeletal muscle cells and other cells among all cell components in different layers of NHO area. **g** The cell type maps show the distribution of FAPs and osteoblasts of NHO sample. **h** IF image shows the PDGFRα^+^ (red) OCN^+^ (green) cells in CDTX-injected muscles from CDTX and NHO groups. Scale bars, 20 μm (*n* = 5). **i** Quantification of the proportion of PDGFRα^+^ OCN^+^ cells in **h** (*n* = 5, unpaired *t*-test). The data are presented as the mean ± s.d. **P < 0.01 compared between groups; *P < 0.05 compared between groups.

**Fig. 3:**
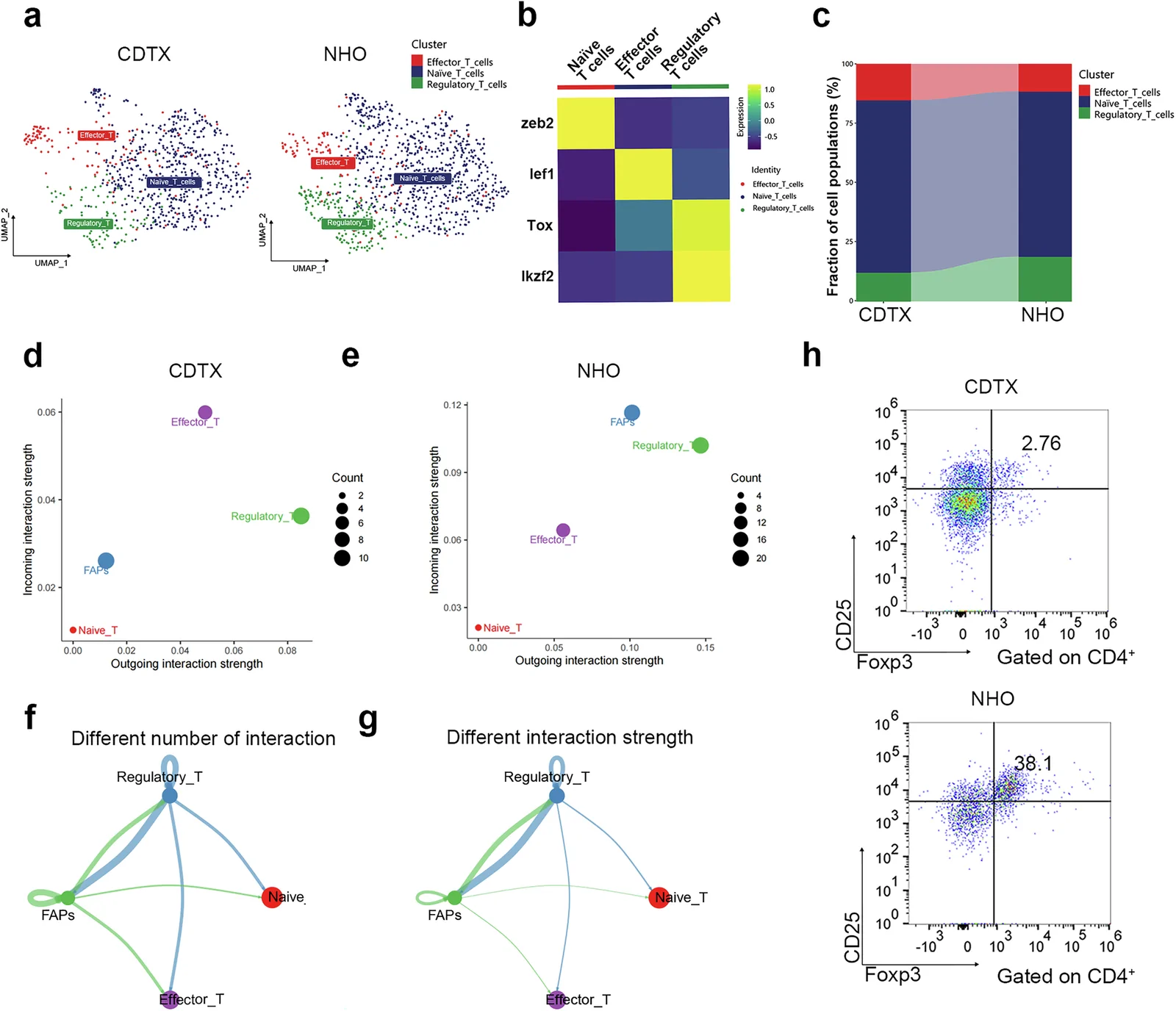
T_reg_ cells enriched in NHO tissue and regulated FAPs. **a** The UMAP plot shows three subclusters of T cells in CDTX and NHO groups. **b** The heat map shows the expression levels of marker genes of T cell subtypes. **c** The stacked bar chart shows the fraction of each T cell cluster in CDTX and NHO groups. **d**,**e** The dot diagram shows the incoming and outgoing strength of FAPs and three T cell subclusters in CDTX (**d**) and NHO (**e**) groups. **f**,**g** The circle plot shows the interaction strength and the number of interactions between FAPs and T cell subtypes in NHO group. **h** The flow cytometry plots depict the frequency of CD25^+^Foxp3^+^ T_reg_ in CDTX and NHO groups.

**Fig. 4:**
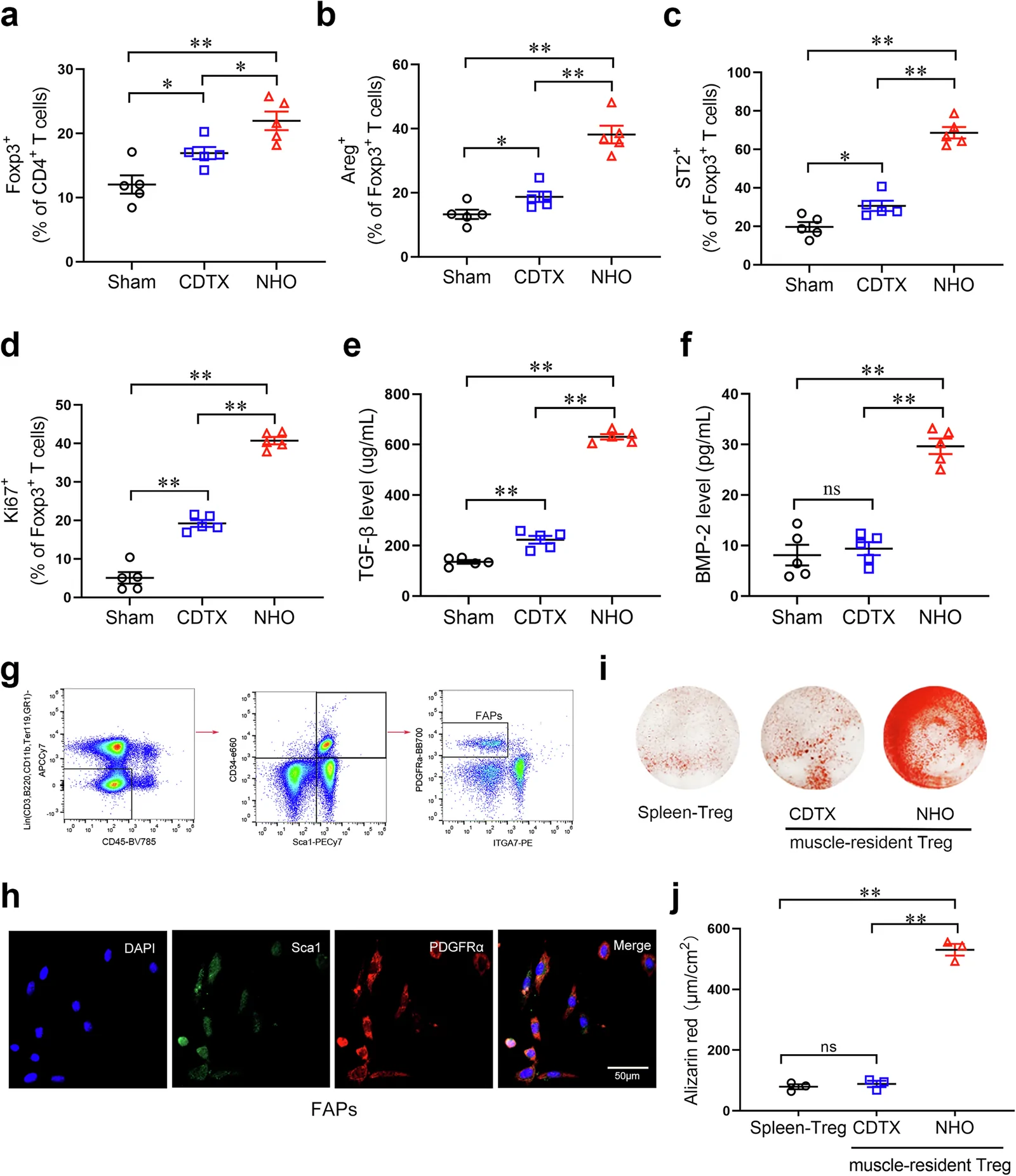
SCI altered the phenotype and functions of T_reg_ in injured muscle. **a–d** Flow cytometry results show the ex vivo characterization of T_reg_ for proliferation: Foxp3^+^CD4^+^ T_reg_ (**a**) Areg^+^Foxp3^+^ T_reg_ (**b**) ST2^+^Foxp3^+^ T_reg_ (**c**) and Ki67^+^Foxp3^+^ T_reg_ (**d**). *n* = 5, one-way ANOVA, Bonferroni post hoc. **e**,**f** ELISA results show the expression level of TGF-β (**e**) and BMP2 (**f**) and in muscle-residing T_reg_ from sham, CDTX and NHO groups. *n* = 5, one-way ANOVA, Bonferroni post hoc. **g** Flow cytometry gating strategy of the isolation of FAPs. **h** IF staining shows PDGFRα^+^ (red) and Sca1^+^ (green) cells isolated from hindlimb muscle of mice. Scale bars, 50 μm. **i** Alizarin Red S staining of FAPs cultured in OM supplemented with CM from spleen T_reg_, CDTX muscle-resident T_reg_ or NHO muscle-resident T_reg_. **j** Quantification of calcium mineralization in i expressed as the mean ± s.d. *n* = 3, one-way ANOVA, Bonferroni post hoc. The data are presented as the mean ± s.d. **P < 0.01 compared between groups; *P < 0.05 compared between groups.

**Fig. 5:**
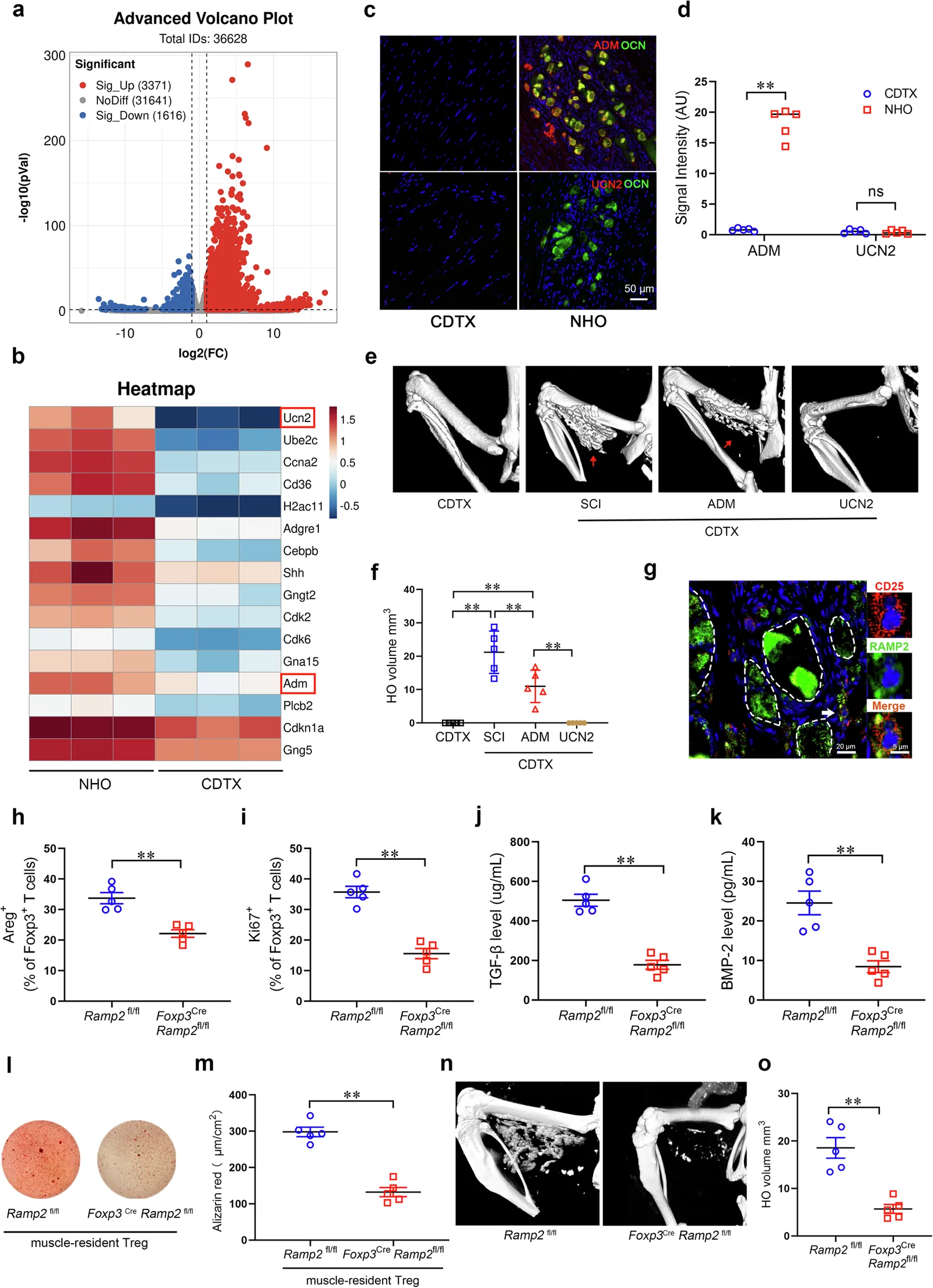
Injured spinal cord-derived ADM induced ossification by expanding muscle-residing T_reg_. **a** The volcano plot shows differentially expressed genes of spinal cord in CDTX versus NHO mice. The *x* axis represents the log_2_ fold change, and the *y* axis represents the −log_10_ *P* value; *n* = 3. **b** The heat map shows the expression levels of 16 spinal cord-secreted proteins in CDTX and NHO groups. **c** IF staining shows the expressions of OCN, UCN2 and ADM. Scale bars, 50 μm. **d** Quantitative analysis of ADM and UCN2 signal intensities in **c**. *n* = 5, one-way ANOVA, Bonferroni post hoc. **e** Representative micro-CT images of CDTX, SCI + CDTX, ADM + CDTX and UCN2 + CDTX groups. **f** Quantitative analysis of HO volumes by micro-CT analysis in **e**. n = 5, one-way ANOVA, Bonferroni post hoc. **g** IF staining shows CD25^+^ (red) and RAMP2^+^ (green) cells in the muscle tissue surrounding NHO sites. Dotted white boxes indicate the area of NHO sites. The white arrow indicates CD25^+^ (red) RAMP2^+^ (green) cells. Right: higher magnification of the CD25^+^ RAMP2^+^ cells shown by the arrow on the left. Scale bars, 20 μm (left), 5 μm (right). **h**,**i** Summary graph for the ex vivo characterization of muscle-residing T_reg_ from *Ramp2*^fl/fl^ and *Foxp3*^Cre^ *Ramp2*^fl/fl^ NHO mice; Areg^+^ Foxp3^+^ T_reg_ (**h**) and Ki67^+^Foxp3^+^ T_reg_ (**i**). *n* = 5, one-way ANOVA, Bonferroni post hoc. **j**,**k** The ELISA assay shows the levels of TGF-β (**j**) and BMP-2 (**k**) in CM of muscle-resident T_reg_ isolated from Ramp2^fl/fl^ and Foxp3^Cre^ Ramp2^fl/fl^ NHO mice. *n* = 5, one-way ANOVA, Bonferroni post hoc. **l** Representative Alizarin Red S staining of FAPs cultured with CM from muscle-resident T_reg_ isolated from *Ramp2*^fl/fl^ and *Foxp3*^Cre^ *Ramp2*^fl/fl^ NHO mice. **m** Quantification of calcium mineralization in **l**. *n* = 5, one-way ANOVA, Bonferroni post hoc. **n** Representative micro-CT images show HO formation in the hindlimb muscles of *Ramp2*^fl/fl^ and *Foxp3*^Cre^ *Ramp2*^fl/fl^ NHO mice. **o** Quantitative analysis of HO volume based on micro-CT images in **n**. *n* = 5, one-way ANOVA, Bonferroni post hoc. The data are presented as the mean ± s.d. ***P < 0.001 compared between groups; ****P < 0.0001 compared between groups.

**Fig. 6:**
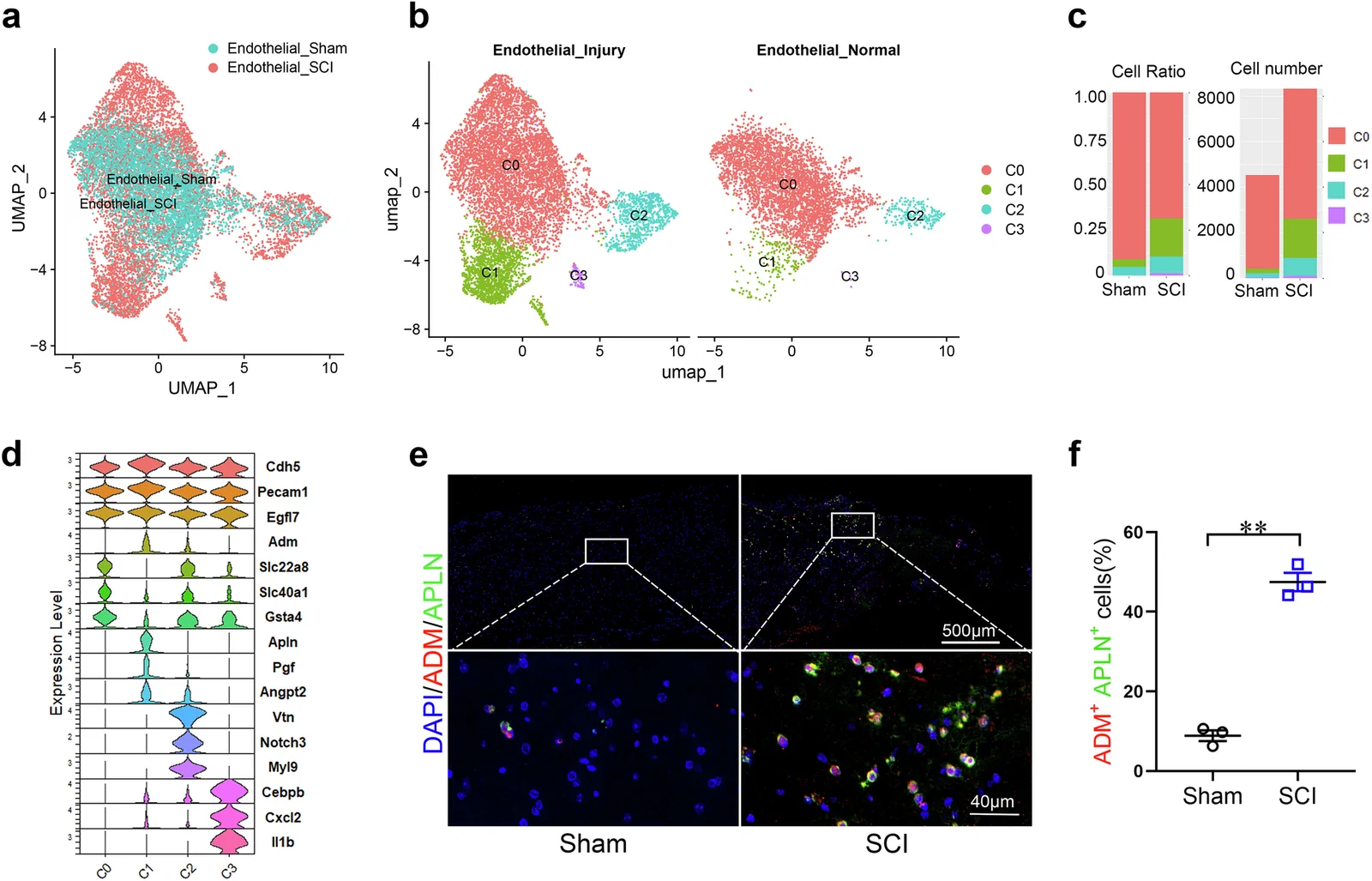
Tip cells in injured spinal cord produced ADM. **a** The UMAP plot shows endothelial cells in spinal cord tissues from sham and SCI group. **b** The UMAP plot shows four subclusters of endothelial cells derived from sham and SCI groups. **c** The stacked bar chart shows the cell number and ratio of each endothelial cell subclusters in spinal cord tissues from sham and SCI groups. **d** The violin plot shows the expression levels of biomarkers for four endothelial cell subclusters. **e** IF staining shows APLN^+^ (green) ADM^+^ (red) cells in sham and the SCI group. Scale bars, 500 μm (top), 40 μm (bottom). **f** Quantitative analysis of the percentage of ADM^+^APLN^+^ cells in **e**. *n* = 3, unpaired *t*-test. The data are presented as the mean ± s.d. **P < 0.01 compared between groups.

**Fig. 7:**
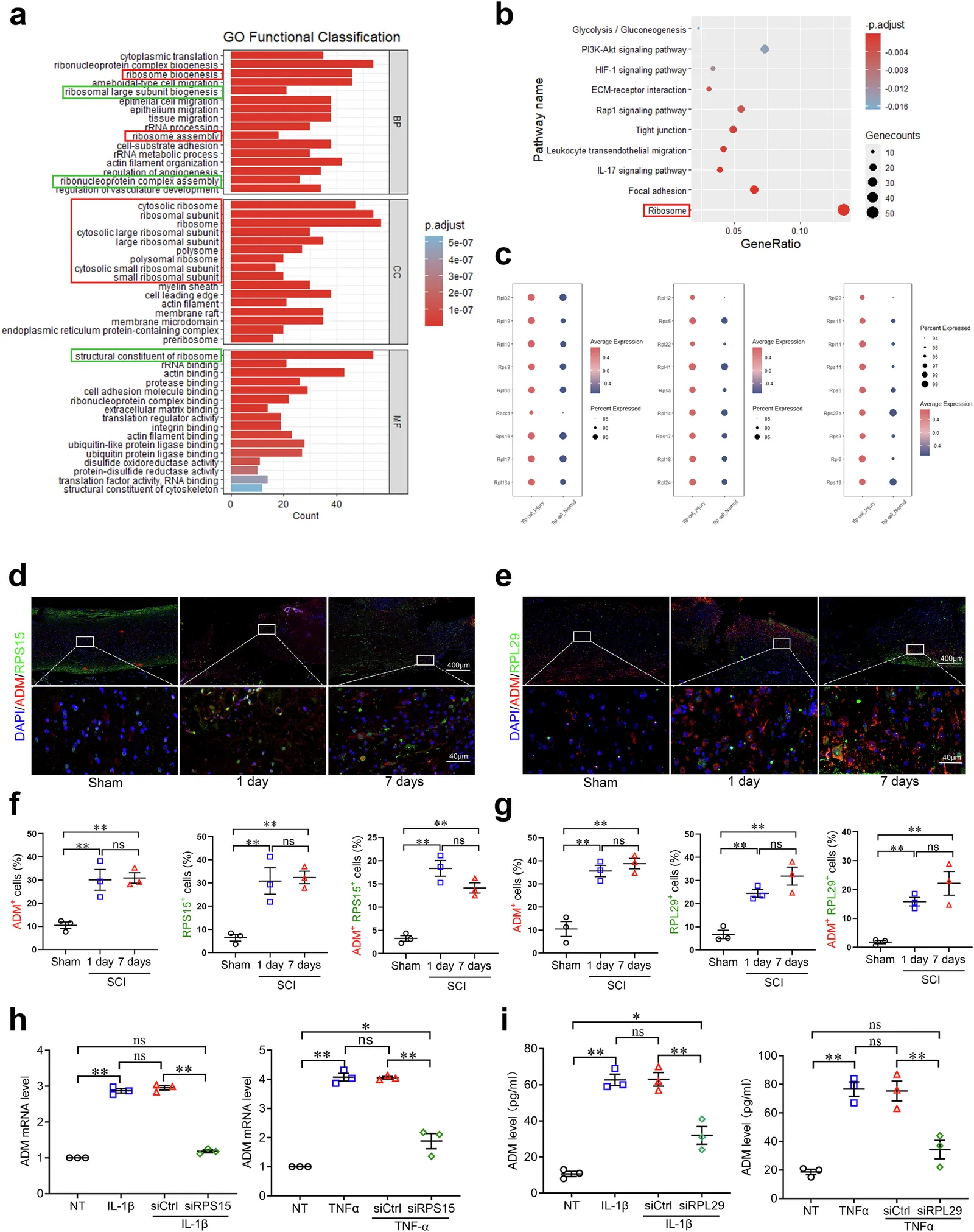
The mechanism of ADM secretion in SCI. **a** The bar chart shows pathway enrichment of GO analysis in tip cells from sham and SCI groups. The red boxes are ribosome-associated enrichment pathways; green boxes are angiogenesis-associated pathways. Horizontal axis shows count (number of genes), and blue to red indicates the adjusted *P* value, bar length indicates the number of genes enriched in a given GO term; molecular function (MF), cellular component (CC) and biological process (BP). **b** The top ten KEGG pathways corresponding to upregulated genes in tip cells after SCI. The red boxes are the ribosomal pathway. The horizontal axis represents the ratio of the number of genes enriched to the target pathway to the total number of target genes in each pathway, and the vertical axis represents the pathway terms enriched; the chromatogram from blue to red represents the adjusted *P* value; the air bubble size represents the number of genes enriched for a given KEGG term. **c** The dot plots show the expression levels of ribosome relative genes in tip cells from injured and normal spinal cord. **d**,**e** IF staining shows the number of ADM^+^ (red) cells and RPS15^+^/RPL29^+^ (green) cells in spinal cord tissue from sham, 1 day post SCI and 7 days post SCI groups. Scale bars, 400 μm (top), 40 μm (bottom). **f** Quantification of ADM^+^ cells, RPS15^+^cells and ADM^+^ RPS15^+^cells in **d**. *n* = 3, one-way ANOVA, Bonferroni post hoc. **g** Quantification of ADM^+^ cells, RPL29^+^cells and ADM^+^RPL29^+^ cells in **e**. *n* = 3, one-way ANOVA, Bonferroni post hoc. **h** Quantitative RT–PCR analysis of ADM mRNA level in tip cells transfected with RPS15 or control siRNAs and stimulated with IL-1β or TNF-α. *n* = 3, unpaired *t*-test. **i** Quantitative ELISA analysis of ADM protein level in tip cells transfected with RPL29 or control siRNAs and stimulated with IL-1β or TNF-α. *n* = 3, unpaired *t*-test. The data are presented as the mean ± s.d. **P < 0.01 compared between groups; *P < 0.05 compared between groups; ns, not significant, with a *P* value >0.05).

**Fig. 8:**
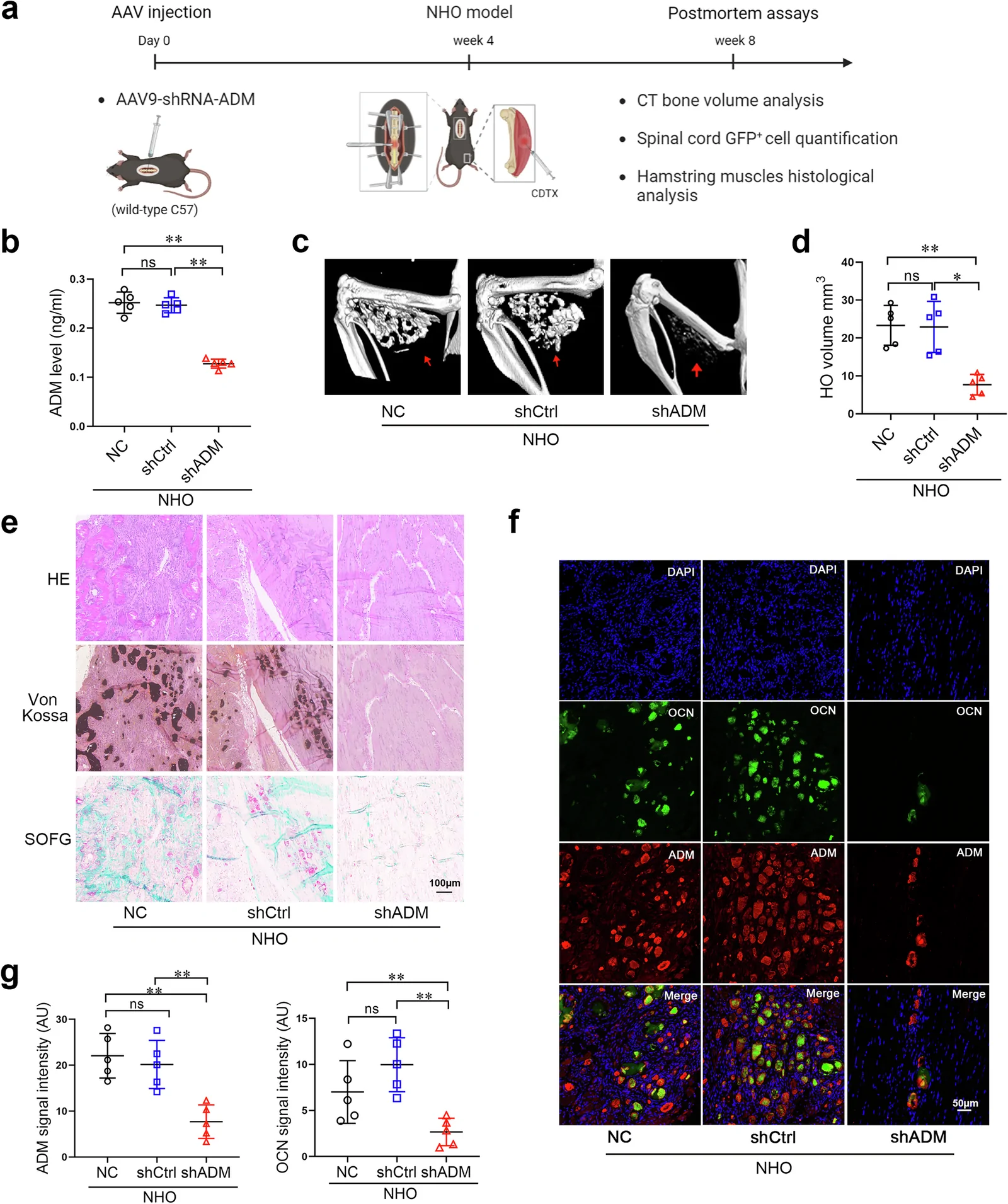
Ablation of spinal cord-derived ADM inhibited the process of NHO. **a** Schematic of intrathecal injection for ADM knockout in the spinal cord of NHO mice. **b** ELISA of ADM concentration in peripheral blood serum of NHO mice in groups of NC, AAV-shCtrl and AAV-shADM. *n* = 5, one-way ANOVA, Bonferroni post hoc. **c** Representative micro-CT images show HO in the hindlimb of NHO mice in groups of NC, AAV-shCtrl and AAV-shADM. The red arrows indicate HO formation. **d** Quantitative analysis of HO volumes by micro-CT analysis in **c**. *n* = 5, one-way ANOVA, Bonferroni post hoc. **e** H&E, Von Kossa, SOFG staining shows HO in the hindlimb of NHO mice in groups of NC, AAV-shCtrl and AAV-shADM. Scale bars, 100 μm. **f** IF staining shows OCN (green) and ADM (red) immunopositivities in the hindlimb of NHO mice in groups of NC, AAV-shCtrl and AAV-shADM. Scale bars, 50 μm. **g** Quantification of ADM and OCN signal intensities in **f**. *n* = 5, one-way ANOVA, Bonferroni post hoc. The data are presented as the mean ± s.d. **P < 0.01 compared between groups; *P < 0.05 compared between groups; ns, not significant, with a *P* value >0.05.

Data obtained from experiments in triplicate and repeated at least three times was represented as mean ± s.d. The unpaired *t*-test was used to compare two groups with Shapiro–Wilk test for normality test. One-way analysis of variance (ANOVA) was performed with Levene’s test for homogeneity of variance, followed by the Bonferroni post hoc test based on the comparison to be made and the statistical indication of each test. Mauchly’s sphericity test was used for sphericity test. For nonparametric data, differences between groups were evaluated with nonparametric Mann–Whitney *U* test, and categorical and binary variables were tested by the Fisher exact test. The exact sample size and the number of independent experiments performed, description of the samples and statistical analyses done were also specified in Figs. 1–8. Statistical significance was accepted at *P* < 0.05. Prism V.8 (GraphPad) was used for statistical analyses and graphs generation.

## Results

### FAPs serve as the original cells of ossification in NHO process

In NHO, destruction of limb muscle fibers occurs concurrently with SCI, where HO typically develops. In the NHO mouse model, as in the human disease, the most affected sites are the spinal cord and limb muscles. To establish the NHO mouse model, we transected the spinal cord of C57BL/6 (B6) mice, followed by intramuscular injection of CDTX into the hindlimbs. No mice with SCI alone or CDTX alone developed NHO, as assessed by micro-CT images (Supplementary Fig. 1a,b). However, the combination of spinal cord transection and CDTX injection induced paralysis accompanied by rigidity of hindlimbs and micro-CT images showed the formation of periarticular nodules (Supplementary Fig. 1a,b). Histology of undecalcified muscles after SCI and CDTX injection demonstrated that the nodules detected by micro-CT were bona fide mineralized bone nodules within the muscle mass, containing osteocytes within lacunae in the bone matrix (Supplementary Fig. 1b,c).

To investigate the cellular origin of ossification in injured muscle tissue following SCI, we collected muscle tissue surrounding the CDTX-injected area from NHO model mice (*n* = 3) and CDTX-injected mice (*n* = 3) for conducting scRNA-seq and spatial transcriptomics sequencing. Reference expression fingerprints of cell types were established using the R package SingleR, with reference to previous studies, to delineate the spatial tomographies of cell type populations in each tissue slide. As a result, 65547 qualified cells were clustered into 13 main cell types using Seurat^20^, including adipocytes (*Cidec*), B cells (*Bank1*), dividing cells (*Top2a*), endothelial cells (*Cdh5*), FAPs (*Pdgfra*), macrophages (*Mrc1*), muscle satellite cells (SCs) (*Pax7*), neurons (*Dpp6*), osteoblasts (*Col1a2*), pericytes (*Rgs5*), Schwann cells (*Cdh19*), skeletal muscle cells (*Klhl31*) and T cells (*Cd247*) (Supplementary Fig. 2a–c). Among them, FAPs accounted for over 10% of the cellular population in NHO group and CDTX group, ranking as the second most abundant cell type after skeletal muscle cells (Supplementary Fig. 2d). Further, separate reclustering of all cells expressing the FAPs marker Pdgfra identified six distinct subclusters (Fig. 1a, b). Subcluster 0, 2 and 3 increased in the NHO group (Fig. 1c). Notably, subcluster 2 exhibited distinct osteogenic potential, marked by high expression of osteogenic marker *Runx2* (Fig. 1d), specific co-expression of *Prrx1* (Fig. 1e) and functional enrichment in osteoblast differentiation pathways (Fig. 1f). It has reported that Prrx1-expressing cells can differentiated into osteogenic progenitors and osteoblasts, resulting in the occurrence of heterotopic bone tissue within injured muscles in response to SCI^21^. Furthermore, reclustering of FAPs populations identified biphenotypic cells expressing multiple mesenchymal and osteoblastic markers. Transcript quantitation in subcluster 2, in comparison with the other subclusters, revealed decreased expression of mesenchymal markers (*Des*, *Dcn* and *Atxn1*) with concomitantly increased expression of osteoblastic markers (*Fgf2*, *Col1a1* and *Col1a2*) and zinc-finger transcription factor (*Zeb1*), which indicated the enhancement of osteogenic differentiation potential^22^ (Fig. 1g). Next, to further corroborate the suggestion that the osteoblast population is trans-differentiation from FAPs, we performed the trajectory analysis, showing multiple differentiation pathway. Pseudotime analysis showed differentiation progressing from FAPs direction to osteoblasts (Fig. 1h).

To systematically characterize the cell compositions and spatial distributions of NHO tissue, we processed freshly frozen hind-limb muscles around NHO area of NHO mouse using 10x Visium Spatial Transcriptomics with four independent capture areas (6.5 mm × 6.5 mm per area) containing a precisely aligned array of 5,000 barcoded spots (55-μm spot diameter, 100-μm center-to-center spacing) (Fig. 2a). We generated spatial transcriptomics datasets from NHO model. In the datasets, there are an average of 71,937 reads and 4,550 genes per spot. We assigned each spot to a specific cell type with the highest probabilistic proportion and the higher expression of classical cell type marker genes in the defined cell clusters, and then, cell components in each spot were analyzed using scRNA-seq data as a reference, resulting in spatial annotation that identified 14 main cell types, including adipocytes, B cells, dividing cells, endothelial cells, FAPs, immunes, macrophages, muscle SCs, neurons, osteoblasts, pericytes, Schwann cells, skeletal muscle cells and T cells (Fig. 2b, c). In addition, we validated the rationale for the cell type annotations applied to the spots in the slide. The tissue slide was spatially segmented into distinct areas including NHO area, femur area and distant area, with these regions identified by pathologists based on H&E staining images (Fig. 2b). Fewer gene expression features were detected in the femur area, indicating that osteogenic activity was primarily observed in the NHO area (Fig. 2b). To comprehensively observe transcriptional landscapes and cell compositions of NHO area, we precisely segmented the NHO areas along the border into seven layers, with each layer representing a 100-µm-wide zone from the border (Fig. 2d). Then we analyzed the features of osteogenesis-related pathways in the seven layers on the ossification area, as well as in the more distant area of muscles, which was defined as distant area. We observed significant enrichment of osteogenic pathways (bone development, collagen-containing extracellular matrix and ossification) within the center of NHO area, with a progressive decline in pathway activity correlating with increasing distance from the ossification center (Fig. 2e). This suggested that the NHO region we defined were undergoing bone formation and remodeling processes. We further analyzed the fractions and features of cell components in the NHO area, finding that the fractions of skeletal muscle cells increased from the center of NHO area to distant area (Fig. 2f). Given that skeletal muscle cells account for more than 75% in the hind-limb muscle samples, we removed the skeletal muscle cells from the specimens to study the trends of other cell types. Among the remaining cells, FAPs were most abundant at the center of the NHO area and decreased toward the outer layers, whereas osteoblasts were primarily concentrated in the first layer (0–100 μm) at the border of the NHO area (Fig. 2f). Furthermore, *Prrx1*^+^ FAPs which has been reported in attending the process of HO were spatially co-localized with osteoblasts (Fig. 2g), and IF staining of mouse hind-limb muscles around NHO area revealed the co-expression of PDGFRα (the marker of FAPs) and OCN (the marker of osteoblasts), further supporting the potential of FAPs to differentiate into osteoblasts (Fig. 2h, i). Therefore, our observations identify FAPs as the primary osteogenic effector cells within the NHO area and drive ectopic bone formation through their differentiation into osteoblasts.

### T_reg_ act as pivotal regulators of FAP fate in NHO

Next, we intended to elucidate the regulatory mechanism of FAPs osteogenesis. Recent studies have highlighted the important roles of immune cells (especially macrophages, T cells and B cells) in muscle repairing and bone remodeling^23,24^. To further explore the inter-cellular communication networks between FAPs and immune cells, we conducted a CellChat analysis using above scRNA-seq dataset. UMAPs analysis revealed T cells, B cells and macrophages in the muscles (Supplementary Fig. 3a). Extensive yet distinct ligand and receptor pairs were revealed between FAPs and macrophages in the CDTX group, while they were specifically identified among FAPs, B cells and T cells in the NHO group. In detail, T cells became the regulator of FAPs in the NHO group, but the regulation of macrophage toward FAPs was decreased in the NHO group (Supplementary Fig. 3b). Bone morphogenetic proteins (BMPs) mediate inter-cellular signaling in bone formation^25^. In the BMP signaling pathway, T cells were the dominant senders while FAPs served as the main receivers (Supplementary Fig. 3c). GO analysis of DEGs in T cells revealed enrichment for terms related to lymphocyte differentiation and regulation of T cell activation (Supplementary Fig. 3d). IF staining confirmed the presence of CD3^+^ T cells in close proximity to PDGFRα^+^ FAPs in NHO samples, whereas CD3^+^ T cells were largely absent in CDTX samples (Supplementary Fig. 3e,f). Collectively, these findings suggest that T cells serve as an important regulator involved in the process of FAPs ossification.

To further identify which T cell subtypes controlling FAPs ossification in response to SCI, we reclustered the T cells from NHO tissue based on their transcriptome profiles. ScRNA-seq analysis revealed three distinct clusters: naïve T cells (*Zeb2*^+^), effector T cells (*Lef1*^+^) and T_reg_ (*Tox*^+^ *Ikzf2*^+^) in CDTX and NHO groups (Fig. 3a, b). In the NHO samples, an increase of the T_reg_ infiltration and a decrease of effector T cells were observed. (Fig. 3c). We compared the outgoing and incoming interaction strength in space, allowing identification of FAPs and T cell subtypes with marked changes in sending or receiving signals. In NHO group, the sending strength of T_reg_ was 1.7-fold compared with CDTX group and both sending and receiving strength of FAPs increased (Fig. 3d, e). Further cellular communication analysis revealed that the modulation from T_reg_ to FAPs was the strongest in NHO tissue (Fig. 3f, g). Flow cytometric analysis of muscle tissue from CDTX and NHO groups confirmed that CD25^+^Foxp3^+^ T_reg_ were markedly increased in NHO group (Fig. 3h). These results indicated that T_reg_ play a regulatory role to FAP fate during the process of NHO.

### Ablation of T_reg_ inhibits HO in SCI mice

Having revealed a unique population of T_reg_ in the injured muscle in response to SCI, we asked whether they directly contributed to NHO. Consequently, we administered anti-CD25 mAb to NHO mice, a strategy for Treg depletion previously used by several studies^26,27^. The results showed that consistent with the observed decrease in ossified FAPs following T_reg_ ablation (Supplementary Fig. 4a,b), NHO formation was markedly diminished in the CDTX-injured muscle tissue (Supplementary Fig. 4c–e). These findings further highlight the pivotal role of T_reg_ in mediating NHO formation.

### NHO muscles harbor expanded muscle-resident T_reg_ that promote the osteogenic differentiation of FAPs through MAPK/p38 pathway

Recent studies have indicated a special phenotype of T_reg_ in the acute injured muscles, known as muscle-resident T_reg_, which play an important role in the control and maintenance of muscle tissue function, homeostasis and integrity by producing several pro-regenerative factors^10^. Our results showed that compared to the sham group, Foxp3^+^ T_reg_ frequencies were increased in the CDTX-injured muscle of both the CDTX (only injected CDTX into the hindlimb of mouse) and NHO groups, with more prominent increase observed in the NHO group (Fig. 4a). Areg and ST2 have been recognized as crucial markers for muscle-resident T_reg_, as Areg plays a critical role in supporting muscle integrity and repair, and ST2 is markedly increased in tissue T_reg_ compared with their lymphoid counterparts^12^. Muscle-resident T_reg_, identified by the expression of Areg or ST2 in Foxp3^+^ Tregs, constituted the majority of increased T_reg_ observed in both the CDTX and NHO groups. Of note, in the NHO group, Areg^+^Foxp3^+^ T_reg_ represented 78.07% ± 5.44% of the Foxp3^+^ T_reg_, while in the CDTX group, they only represented 38.17% ± 6.18% (Fig. 4b). ST2^+^Foxp3^+^ T_reg_ in the NHO group accounted for 68.66% ± 6.62%, compared with 30.64% ± 5.94% in the CDTX group (Fig. 4c). These results suggest that SCI has powerful potential to induce much more muscle-resident T_reg_ in injured muscles. Furthermore, the analysis of the proliferative marker Ki67 revealed an increase in muscle-resident T_reg_ in both the NHO and CDTX groups compared with the sham group. Likewise, this upward trend was markedly more pronounced in the NHO group than in the CDTX group (Fig. 4d), suggesting that SCI enhances a specific expansion of muscle-resident T_reg_ in injured muscle tissue. In addition, BMP-2 and TGF-β, two kinds of classical osteogenic factors, were found to be secreted at higher levels by muscle-resident T_reg_ from NHO group, while they were seldom produced by muscle-resident T_reg_ from sham and CDTX groups (Fig. 4e, f).

To confirm the osteogenic capacity of muscle-resident T_reg_ to FAPs, FAPs were successfully collected from normal mouse muscle tissue (Fig. 4g, h) and were cultured with conditional medium (CM) collected from spleen T_reg_, CDTX muscle-resident T_reg_ or NHO muscle-resident T_reg_. Alizarin Red S staining indicated that only CM from NHO muscle-resident T_reg_ induced FAP mineralization (Fig. 4i, j). These results indicate that muscle-resident T_reg_ in NHO acquire an enhanced pro-osteogenic secretory function that promotes the osteogenic differentiation of FAPs. To further delineate the intracellular signaling pathway activated in FAPs downstream of muscle-resident T_reg_-derived osteogenic factors, we performed KEGG analysis of DEGs in FAPs from NHO and CDTX-injured tissues. The MAPK signaling pathway was significantly enriched in FAPs from NHO group, suggesting that MAPK signaling may participate in the osteogenic conversion of FAPs during NHO formation (Supplementary Fig. 5a). Several studies have reported that MAPK-dependent pathways, with p38, ERK and JNK, were involved during the process of osteogenic differentiation^28–30^. To test this experimentally, FAPs were isolated from muscle tissues of CDTX and NHO groups and observed phosphorylation of p38, ERK and JNK expressions. The results showed that only phosphorylation of p38 was significantly increased in FAPs of NHO compared with CDTX group (Supplementary Fig. 5b,c). We therefore focused on the p38 MAPK pathway and found that pharmacological inhibition of p38 MAPK with SB203580 attenuated BMP-2- and TGF-β-induced Runx2 expression at both the mRNA and protein levels (Supplementary Fig. 5d–f). Functionally, Alizarin Red S staining showed that MAPK/p38 inhibition significantly attenuated calcium deposition in FAPs induced by BMP-2 and TGF-β (Supplementary Fig. 5g,h). These results indicate that BMP-2 and TGF-β, two osteogenic factors enriched in NHO-associated muscle-resident T_reg_, can promote FAP osteogenic conversion through MAPK/p38 signaling. Taken together, these findings indicate that SCI promotes the accumulation and pro-osteogenic functional activation of muscle-resident T_reg_ in injured muscle. Through the secretion of osteogenic factors such as BMP-2 and TGF-β, muscle-resident T_reg_ activate MAPK/p38 signaling in FAPs, thereby driving Runx2 expression and FAP osteogenic differentiation.

### Spinal cord-derived ADM promotes the expansion and pro-osteogenic activation of muscle-resident T_reg_

NHO developed exclusively in the CDTX-injured muscle of SCI mice, which suggested that spinal cord-derived modulator is indispensable in NHO formation (Supplementary Fig. 1a–c). To investigate the potential regulatory factors leading to NHO formation after SCI, spinal cord tissues were collected from CDTX mice (*n* = 3) and NHO mice (*n* = 3) 3 days post surgery for bulk RNA sequencing. The results revealed 4,987 differentially expressed genes, with 3,371 genes upregulated and 1,616 genes downregulated (fold change >1.5; *P* < 0.05) in spinal cord between CDTX and NHO groups (Fig. 5a). These results indicate a substantial alteration in the spinal cord gene networks of NHO mice. Among these upregulated genes, 16 encoded secreted proteins that were enriched in the NHO group. When filtered for factors encoding neuroactive ligands, the candidates remaining were *Adm* and *Ucn2* (Fig. 5b). IF staining showed that ADM was aberrantly upregulated in the NHO sites, whereas the expression of *Ucn2* was not different from that in the CDTX-injected sites and NHO sites (Fig. 5c, d). To further confirm the effect of ADM on the NHO formation, we used micro-CT to observe the formation of HO. The results demonstrated that osteophyte formation was observed in the mice limbs with the co-injection of ADM and CDTX, whereas it was not induced by the co-injection of UCN2 and CDTX (Fig. 5e, f). Furthermore, muscle samples were collected from NHO patients, only patients with SCI and only fracture patients with HO (named as traumatic HO (THO)) and only fracture patients. Radiological examination and histological staining confirmed the HO formation in patients with THO and NHO but not in patients with only SCI or only fracture (Supplementary Fig. 6a,b). IF staining revealed that ADM was aberrantly upregulated in the muscle tissue from both the patients with THO and NHO, with a particularly notable increased in the patients with NHO, but it was seldom increased in patients with only SCI or only fracture (Supplementary Fig. 6c,d). Similar to the IF staining results, the serum level of ADM was elevated in both the THO and NHO patients, with a particularly notable increased in the NHO patients (Supplementary Fig. 6e). The clinical baseline characteristics, including age, sex, injury time and NSAID use, were comparable among the groups. Importantly, Pearson correlation analysis demonstrated that serum ADM levels were positively correlated with CT-measured HO volume in patients with NHO (*r* = 0.8817, *P* = 0.0017, 95% confidenct interval (CI) 0.5249 to 0.9749). By contrast, although a positive trend was also observed in patients with THO, the correlation did not reach statistical significance (*r* = 0.5618, *P* = 0.0573, 95% CI −0.0179 to 0.8588) (Supplementary Table 4). These findings support a clinically relevant association between circulating ADM and NHO burden in humans.

Based on the above analysis, we hypothesized that the injured spinal cord produces a substantial amount of ADM, which is transported to the periphery tissue to promote the osteogenic differentiation of FAPs. To validate this hypothesis, various concentrations of ADM (0.1–10 nM) were added into osteogenic medium to stimulate FAPs. However, the results of Alizarin Red S staining indicated that ADM failed to enhance the osteogenic differentiation of FAPs (Supplementary Fig. 7a,b). RT–qPCR analysis showed that the mRNA level of the osteogenic marker Bglap in FAPs did not increase under ADM stimulation at different concentrations. (Supplementary Fig. 7c). The IF staining results showed that T_reg_ infiltrated around the ossified area and expressed the ADM specific receptor domain protein, receptor activity-modifying protein 2 (RAMP2) (Fig. 5g), which implied the interaction between ADM and T_reg_ in NHO sites. To further determine whether RAMP2 in muscle-resident T_reg_ is required for ADM-mediated NHO formation in vivo, we generated T_reg_-specific *Ramp2*-conditional-knockout mice (*Foxp3*^Cre^*Ramp2*^fl/fl^). *Ramp2*^fl/fl^ mice were used as controls. Compared with *Ramp2*^fl/fl^ control mice, Areg and Ki67 expression were significantly reduced in Foxp3⁺ T_reg_ isolated from injured muscle tissue of *Foxp3*^Cre^*Ramp2*^fl/fl^ mice (Fig. 5h, i), indicating impaired activation and proliferative capacity of muscle-resident T_reg_. Moreover, *Ramp2* deletion in T_reg_ markedly reduced the production of the osteogenic factors BMP-2 and TGF-β (Fig. 5j, k). Consistently, conditioned medium from *Ramp2*-deficient muscle-resident T_reg_ markedly reduced osteogenic differentiation of FAPs in vitro, as evidenced by decreased Alizarin Red S staining (Fig. 5l, m). Importantly, micro-CT analysis further showed that NHO formation was substantially attenuated in *Foxp3*^Cre^*Ramp2*^fl/fl^ NHO mice compared with control mice (Fig. 5n, o). These findings provide further evidence that RAMP2 in T_reg_ is required for ADM-mediated osteogenic programming of muscle-resident T_reg_ and subsequent NHO formation in vivo. Consistent with these genetic findings, pharmacological blockade of ADM receptor with AMA also resulted in a reduction of muscle-resident T_reg_ in the NHO muscle tissue (Supplementary Fig. 8a,b). In vitro, ADM stimulation enhanced the secretion of the osteogenic factors BMP-2 and TGF-β by muscle-resident T_reg_, whereas AMA markedly attenuated this effect (Supplementary Fig. 8c,d). The Alizarin red staining results showed that ADM alone failed to induce FAP mineralization, whereas conditioned medium from ADM-treated muscle-resident T_reg_ markedly promoted calcium deposition in FAPs. By contrast, conditioned medium from T_reg_ treated with ADM together with AMA showed a reduced ability to promote FAP mineralization (Supplementary Fig. 8e,f). Furthermore, micro-CT analysis showed that AMA significantly attenuated HO formation in NHO mice (Supplementary Fig. 8g,h).

Collectively, these results indicate that ADM does not directly induce FAP osteogenic differentiation. Instead, ADM signals through RAMP2 on muscle-resident T_reg_ to promote the expansion of muscle-resident T_reg_ and enhance their pro-osteogenic phenotype, characterized by increased BMP-2 and TGF-β production, thereby driving FAP osteogenic differentiation and subsequent NHO formation.

### ADM is derived from tip cells in the injured spinal cord

To illustrate the source of ADM protein, we accessed a publicly available scRNA-seq dataset from the injured spinal cord (GSE162610)^31^. Gene expression data were aligned to be projected in a two-dimensional space through UMAP, and 13 clusters were identified, including microglial cells (*Cx3cr1*), neutrophils (*Wfdc17*), endothelial cells (*Pecam*1), astrocytes (*Sox9*), oligodendrocyte precursor cells (*Cspg5*), monocytes (*Mmp3*), myeloid cells (*Top2a*), mural cells (*Myl9*), oligodendrocytes (*Fa2h*), natural killer T cells (NKTs) (*Agt*), fibroblasts (*Col1a1*), neurons (*Scg2*) and lymphocytes (*Cd2*) (Supplementary Fig. 9a–c). Injured spinal cord exhibited altered abundances of oligodendrocytes, oligodendrocyte precursor cells, endothelial cells, astrocytes and microglial cells (Supplementary Fig. 9d). Among these clusters, *Adm* was mainly expressed in endothelial cells (Supplementary Fig. 9e). Then, we performed further cluster analysis of endothelial cells in the spinal cord. According to previous studies^31,32^, we identified four distinct subtypes of endothelial cells in spinal cord (Fig. 6a, b). Notably, both the number and ratio of cells in cluster 1 increased in the SCI group (Fig. 6c). The highest DEGs provides a unique molecular signature for each kind of endothelial cell. The C1 endothelial cells were identified as tip cells based on their expression of the canonical marker *Apln* and *Angpt2* (Fig. 6d). As we know, tip cells play a guiding role in angiogenesis and vasculogenesis, especially during inflammatory responses, where they are crucial for directing the formation of new blood vessels^33,34^. IF staining demonstrated that tip cells in the injured spinal cord exhibited a significantly increased expression of ADM (Fig. 6e, f). Collectively, a distinct population of endothelial tip cells is initially observed in the vicinity of the SCI site, which is responsible for the production of ADM.

### Inflammation promotes Tip cells to secrete ADM through ribosomal activation

We further investigated the downstream signaling pathway involved in producing ADM in tip cells. GO analysis and KEGG analysis of scRNA-seq dataset (GSE162610)^31^ revealed that ribosomal activity pathways were significantly upregulated in tip cells from injured spinal cord tissue (Fig. 7a, b). In addition, ribosome relative genes were also significantly increased in tip cells from injured spinal cord tissue (Fig. 7c). Ribosomal protein S15 (RPS15, 40S subunit) and ribosomal protein L29 (RPL29, 60S subunit) are essential structural components of ribosome. Both play crucial roles in protein synthesis. The IF staining results showed that at 1 day and 7 days post SCI, the expression of ADM and ribosomal proteins (RPS15 and RPL29) were significantly increased in the injured spinal cord tissue, and the proportion of cells that co-expressed both ADM and ribosomal proteins (RPS15 or RPL29) were also elevated (Fig. 7d–g). To further investigate the mechanism by which tip cells secrete ADM, hUVECs were treated with VEGF gradient (0–25 ng/ml) to differentiate into tip cells according to our previous publication^17^. Multiple inflammatory mediators were involved in the complex inflammatory micro-environment of SCI, among which IL-1β and TNF-α are the most consistently reported pro-inflammatory cytokines in this setting. Thus, we chose these two factors to mimic the inflammatory condition of the injured spinal cord in vitro^35,36^. These representative inflammatory stimuli significantly increased the production of ADM from tip cells, and knockdown of *RPS15* and *RPL29* of tip cells (siRNA sequence is presented in Supplementary Table 3) led to a significant reduction in ADM (Fig. 7h, i). These results demonstrate that SCI-related inflammatory signals induce ribosome-dependent ADM biosynthesis in tip cells.

### Ablation of spinal cord-derived ADM inhibits muscle ossification

To further investigate the impact of ablation of spinal cord-derived ADM on the occurrence of NHO, we constructed AAV-shCtrl (enhanced green fluorescent protein (eGFP) co-expression) and AAV-shADM (eGFP co-expression) vectors and respectively intrathecal transfected them into the spinal cord of NHO mice, and then constructed the NHO mouse model as before at 4 weeks after intrathecal transfected of AAV. Finally, at 8 weeks after intrathecal injection, we traced the ADM and evaluated the bone volume in muscle tissue (Fig. 8a and Supplementary Fig. 10a–c). GFP^+^ cells were found throughout the spinal cord (Supplementary Fig. 10d), and transfection of AAV-shADM resulted in a significant reduction of ADM in the spinal cord (Supplementary Fig. 10e–g). Correspondingly, ELISA assay showed a significant reduction in the serum ADM of NHO mice after the knockdown of their spinal cord-derived ADM (Fig. 8b). Micro-CT analysis revealed a significant decrease in the volume of heterotopic bone in NHO mouse model by knockdown of the spinal cord-derived-ADM (Fig. 8c, d). Similarly, HE, Von Kossa and SOFG staining also showed an obvious reduction of bone formation in injured muscle tissue (CDTX injection area) of NHO mice with the knockdown of spinal cord-derived-ADM (Fig. 8e). To further examine the importance of ADM in new bone formation, we performed IF staining for osteoblast specific marker OCN. The results showed that ADM co-localized with OCN expression sites in CDTX-injured muscle tissue of NHO mice, and after knockdown of spinal cord-derived-ADM, OCN signal intensity was significantly reduced following the decrease of ADM signal intensity (Fig. 8f, g). Together, these findings strongly suggest that spinal cord-derived-ADM induced the HO in the injured muscle tissue, and it is an effective target for inhibiting NHO.

## Discussion

Although it is generally agreed that NHO is triggered by concurrent central and peripheral injuries^37^, the fundamental question of how SCI remotely instigates heterotopic bone formation remains unanswered. Dissecting these long-range cellular and molecular events is therefore essential, not only to develop novel therapies but also to illuminate the general principles of CNS-led control over peripheral tissue homeostasis and pathology.

Through the first application of scRNA-seq and spatial transcriptomics to this problem, our study defines a concrete neuro–immune–bone axis. It is revealed that CNS injury upregulates the neuropeptide ADM in tip cells, which then escapes the compromised blood–brain barrier and systemically targets injured muscle. ADM then promotes the proliferation and functional conversion of muscle-resident T_reg_, which are responsible for driving FAPs to undergo osteogenic differentiation and ultimately form NHO.

SCI induces not only local damage but also systemic multi-organ dysfunction by disrupting brain-spinal cord communication^38^. This results in loss of motor, sensory, and autonomic functions, activating widespread inflammation and immune system irregularities^39^. We observed that neither isolated intramuscular injection of CDTX nor spinal cord transection alone led to the development of HO, which suggests that local changes at the injury site following SCI determine the HO in the muscle. The inflammatory milieu after SCI is highly complex and involves multiple pro-inflammatory cytokines. Previous studies have shown that IL-1β and TNF-α are rapidly induced after SCI and represent canonical inflammatory mediators in the injured spinal cord. Therefore, in our in vitro system, IL-1β and TNF-α were selected as representative SCI-associated inflammatory cues to model the inflammatory environment of the injured spinal cord, rather than as exclusive upstream triggers of ADM production^35,36,40,41^. Under these representative inflammatory conditions, tip cells exhibited increased ADM production through ribosomal activation, supporting the idea that SCI-associated inflammatory cues can promote pathological ADM biosynthesis in tip cells. It is crucial to distinguish this pathological ADM output from its physiological role: under normal conditions, ADM derived from conventional sources such as endothelial cells maintains homeostasis and exerts protective activities^15,42,43^. In the context of SCI, however, the injured spinal cord tip cells generate an abnormal ADM output that contributes to the elevated circulating ADM pool. This centrally derived ADM subsequently acts as a pathological signal on peripheral muscle-resident T_reg_, promoting their pro-osteogenic activation and disrupting peripheral tissue homeostasis.

The pathological importance of this tip cell-ADM axis is further highlighted by its misappropriation of a physiological osteogenic program. Previous study of human embryonic bone formation found that tip cells promote osteoblastic differentiation, osteocyte mineralization and osteoclast recruitment in the maturing bone^44^ and were predicted to improve differentiation of postnatal perivascular osteoprogenitors^45^. Our findings demonstrate that SCI pathologically co-opts this inherent osteogenic capacity of tip cells to drive ectopic bone formation. Substantiating the pivotal role of this axis, we found ADM, the key mediator secreted by tip cells, to be significantly upregulated in the peripheral blood and muscle tissue of both NHO mice and patients. In conclusion, our study confirms that SCI-induced inflammation promotes tip cells to secrete ADM, which subsequently accumulates at the injured muscle site, where it functions not as a homeostatic regulator but as a key mediator of NHO.

It is well established that in the injured muscle tissue, myofibers is generated to facilitate repair. However, whether ADM result in ossification instead of generating functional myofibers following SCI remains a fascinating stem cell biology question. Our study finds that muscle-resident T_reg_ of NHO mouse model highly expressed the receptor of ADM after SCI. Previous study has reported in the arthritis mouse model, ADM can induce T_reg_ expansion^16^. Besides, expression of ADM and its receptor have also been observed in osteoblasts during later stages of rodent embryogenesis^46^. Further research has demonstrated that ADM acts on osteoblasts to increase cell growth and increases protein synthesis in vitro and the area of mineralized and unmineralized bone in vivo^47^. Our results indicate that under SCI, ADM mediates ossification not by acting on osteoprogenitors directly but by acting on muscle-resident T_reg_ through RAMP2 to expand muscle-resident T_reg_ and enhance their pro-osteogenic phenotype.

Traditionally known for their immunomodulatory roles, T_reg_ also play a pivotal part in tissue repair and homeostasis^24^. This is particularly evident in skeletal muscle, where a specialized population of muscle-resident T_reg_ is essential for regeneration following acute and chronic injury^10,48^. These cells can directly influence local parenchymal and mesenchymal cells at the injury site^24^. A key mechanism involves their interaction with FAPs; for instance, it has been suggested that FAPs and T_reg_ may communicate via the IL-33 signaling pathway to promote muscle repair^10^. Notably, muscle-resident T_reg_ highly express Areg, a factor crucial for their regenerative function^12^. This foundational role in tissue homeostasis positions muscle-resident T_reg_ as a critical cellular substrate that can be susceptible to pathological directives from the systemic milieu. Our study demonstrates that following SCI, spinal cord-derived ADM acts on muscle-resident T_reg_ through RAMP2, promoting their expansion and enhancing their pro-osteogenic phenotype characterized by increased BMP-2 and TGF-β production, thereby driving FAP osteogenic differentiation and contributing to NHO formation.

Generally speaking, FAPs have the ability to differentiate into fibroblasts and adipocytes, playing a beneficial role in supporting muscle repair and regeneration^5,49^. SCs are another population of myogenic stem cells residing within the myofiber under the myofiber basal lamina^50^. Both two populations of stem/progenitor cells of adult skeletal muscles have osteogenic potential. It has been reported that in FOP models, mutations in the *ACVR1* gene result in hypersensitivity of the receptor to BMP signaling, and the consequent over-activation of the ACVR1 receptor drives FAPs to differentiate into osteoblasts^51^. Although SCs could mediates the β-catenin signaling pathway in the repair of osteoporotic fracture, maintaining themself differentiation into osteoblasts and restricting osteoclastogenesis^52^. As SCs and FAPs respectively exert their osteogenic potential through different signal pathway under different disease conditions, identifying the dominant cellular mediator through which the injured CNS disrupts peripheral tissue fate to form NHO remains a critical and unresolved question. From the perspective of the cells of origin of NHO, Hsu-Wen Tseng et al. confirmed that following muscle injury, SCI causes the up-regulation of PDGFRα expression on FAPs but not SCs^21^. In addition, SCs fail to regenerate myofibers in the injured muscle, while muscle-resident FAPs exhibit reduced apoptosis and continued proliferation, allowing them to undergo osteogenic differentiation into NHO^21^. Consistent with these findings, our research found that the frequency of SCs did not increase in NHO sites. With the technology of single-cell RNA sequencing and spatial transcriptomics, our results suggest that Prrx1^+^ FAPs serve as the primary cell-of-origin for NHO. Collectively, we observed a marked increase in communication between FAPs and muscle-resident T_reg_ in NHO group. We further found that muscle-resident T_reg_ with the situation of ADM secreted higher levels of osteogenic factors to induce osteogenic differentiation of FAPs. Our additional mechanistic data further link this muscle-resident T_reg_-derived osteogenic signal to the intracellular osteogenic program of FAPs, showing that TGF-β and BMP-2 activate MAPK/p38-Runx2 signaling to promote FAP osteogenic conversion and mineralized matrix formation.

From a therapeutic perspective, the ADM–T_reg_–FAP axis identified in this study provides potential intervention points for NHO after SCI. Because spinal cord-derived ADM acts through RAMP2 to promote the expansion and pro-osteogenic activation of muscle-resident T_reg_, blockade of ADM signaling or ADM-RAMP2-dependent T_reg_ activation may help interrupt the pathological communication between the injured spinal cord and peripheral muscle. In addition, targeting downstream T_reg_-FAP crosstalk may represent a more distal strategy to suppress FAP osteogenic conversion. However, these therapeutic strategies require careful consideration. ADM has important physiological roles in vascular homeostasis, vasodilation, endothelial protection and tissue repair, whereas T_reg_ are essential for immune tolerance and muscle regeneration. Therefore, broad systemic inhibition of ADM signaling or global suppression of T_reg_ function may cause unwanted vascular or immune-related side effects. Future therapeutic approaches should therefore aim for temporally controlled, tissue-selective or pathway-specific modulation of the pathological ADM–T_reg_–FAP axis, rather than broad suppression of ADM or T_reg_ function. Furthermore, our clinical analysis showed that serum ADM levels were moderately correlated with CT-based HO volume (*r* = 0.8817, *P* = 0.0017), suggesting that circulating ADM may have potential value for identifying patients at high risk of NHO or monitoring disease activity, although this possibility requires validation in larger clinical cohorts.

In conclusion, our study elucidates a definitive neuro–immune–bone axis that mediates heterotopic ossification following spinal cord injury. We demonstrate that the neuropeptide ADM, upon reaching the peripheral muscle, acts as a critical pathological signal that activates muscle-resident T_reg_, which in turn redirect the fate of FAPs from regeneration toward osteogenesis. More broadly, the current study provides a mechanistic paradigm for how the central nervous system exerts long-range control over the peripheral skeletal system. By identifying ADM and its downstream cascade as a specific communication channel from the injured CNS to the muscle, we establish a fundamental principle of remote neural control of tissue homeostasis. Consequently, this research not only identifies the neuro–immune–bone axis as a promising therapeutic target for NHO but also provides a foundational framework for understanding how neurological damage can systemically recalibrate tissue fate and integrity across the organism.

## Supplementary information

Supplementary Information
