# Supplementary material for: Adrenomedullin from spinal cord injury drives heterotopic ossification in skeletal muscle via a neuro–immune–bone axis
Source: Exp Mol Med. 2026 Sep 7;58(9):3042–60. doi: 10.1038/s12276-026-01836-7 (PMC13633261; doi:10.1038/s12276-026-01836-7)
Supplement: Supplementary file 1 [file 12276_2026_1836_MOESM1_ESM.pdf]

Supporting Information

Adrenomedullin from Spinal Cord Injury Drives Heterotopic Ossification in Skeletal Muscle via a Neuro-Immune-Bone Axis

Supplementary Figure 1. The successful construction of NHO mouse model

Supplementary Figure 2. The single-cell landscape of CDTX-injected muscle tissue from CDTX and NHO mice

Supplementary Figure 3. The inter-cellular communication between T cells and FAPs

Supplementary Figure 4. Treg depletion significantly impedes ossification of FAPs in the injured muscle after SCI

Supplementary Figure 5. Muscle-resident Treg-derived osteogenic factors activate MAPK/p38 signaling in FAPs to promote osteogenic differentiation

Supplementary Figure 6. ADM was increased in THO and NHO patients

Supplementary Figure 7. ADM alone failed to induce osteogenic differentiation of FAPs *In vitro* experiments

Supplementary Figure 8. Pharmacological blockade of ADM/RAMP2 signaling attenuates the pro-osteogenic function of muscle-resident Tregs

Supplementary Figure 9. ScRNA-seq showed the profile of spinal cord of SCI and sham groups

Supplementary Figure 10. Validation of infection efficiency of AAV-shADM

Supplementary Table 1. Primers for RT-qPCR analysis of gene expression

Supplementary Table 2. Reagent or resource

Supplementary Table 3. List of RNAi sequences

Supplementary Table 4. Clinical characteristics of human subjects

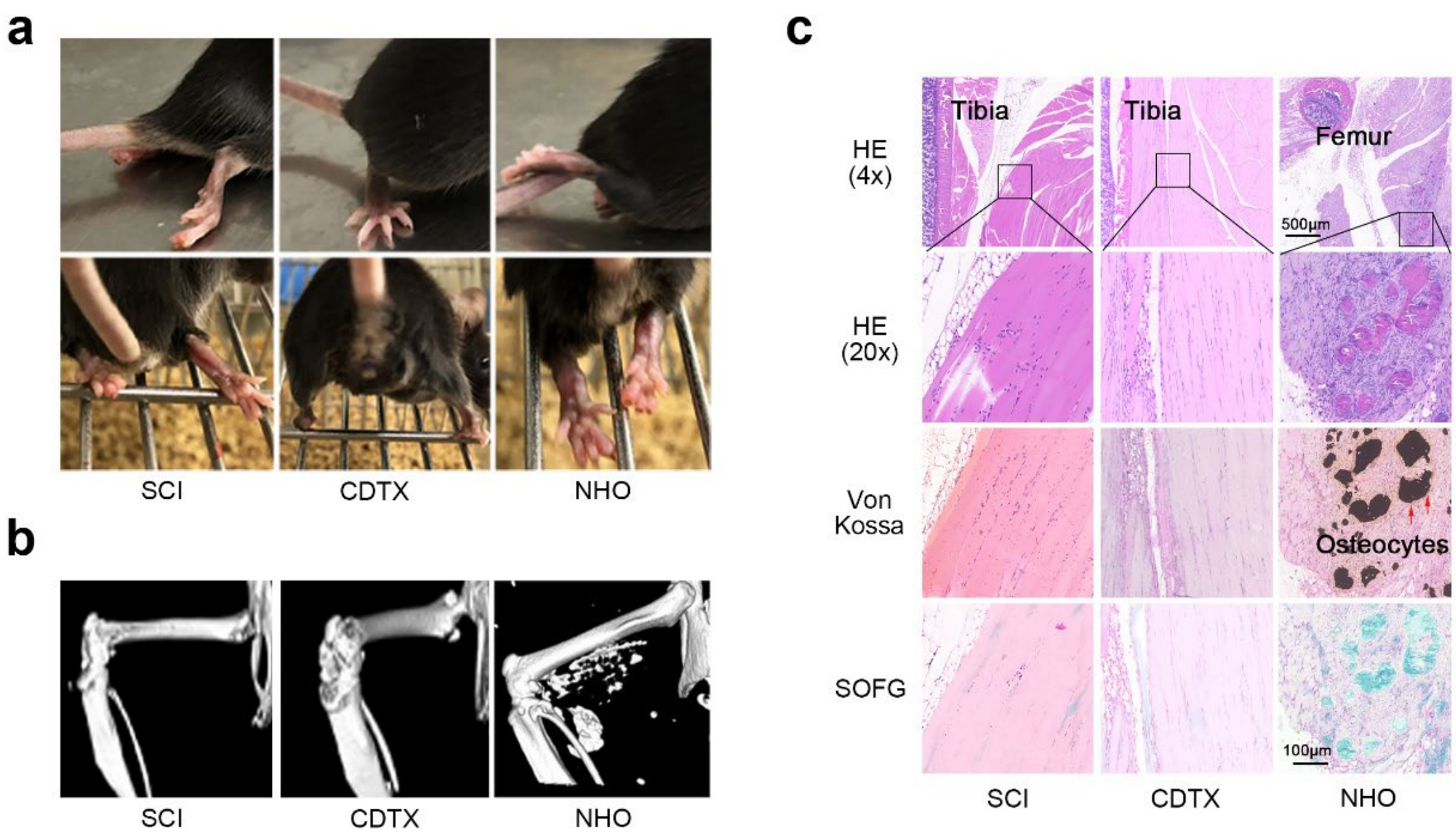

Supplementary Figure 1. The successful construction of NHO mouse model.

a. Behavioral character images showed hindlimb movement of mice in SCI, CDTX and NHO (SCI + CDTX) groups.

b. Micro-CT three-dimensional reconstructed images showed HO in the hindlimb of mice in SCI, CDTX and NHO groups.

c. Hematoxylin-eosin (HE), Von Kossa and Safranin O/Fast Green (SOFG) staining of HO in the hindlimb of mice in SCI, CDTX and NHO groups. Red arrows indicated the osteocytes. Scale bars: 500µm (upper), 100µm (middle and lower).

SCI: SCI surgery plus intramuscular (i.m.) injection of saline; CDTX: sham surgery plus i.m. injection of CDTX; SCI + CDTX: SCI plus i.m. CDTX injection.

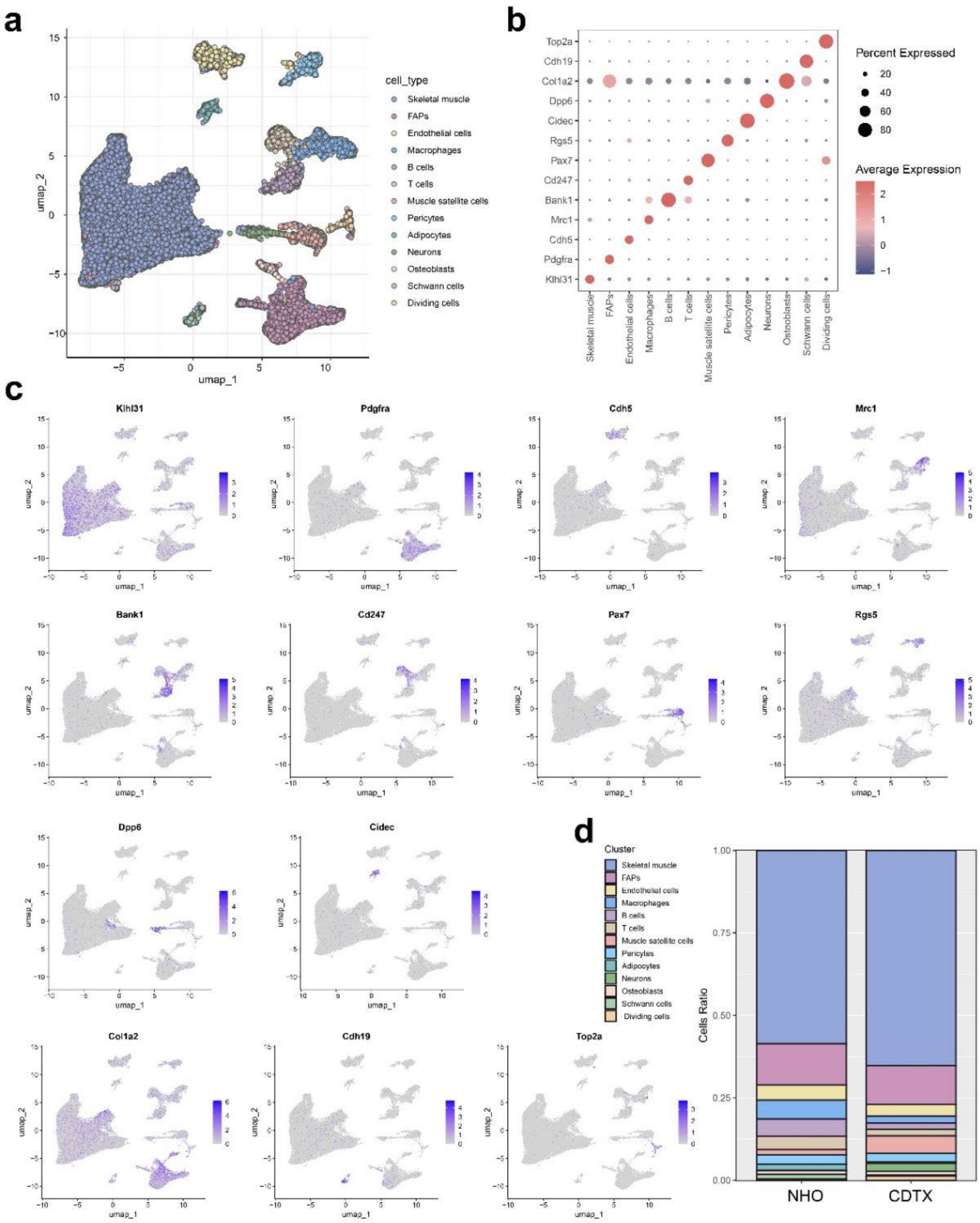

Supplementary Figure 2. The single-cell landscape of CDTX-injected muscle tissue from CDTX and NHO mice.

a. The UMAP plot of 65547 qualified cells from all samples (NHO, n=3; CDTX, n=3), displaying 13 major cell types.

b-c. The heatmap and UMAP plot showed the expression level of markers for 13 major cell types.

d. The stacked bar chart showed the cell ratio of 13 major cell types in NHO group and CDTX group.

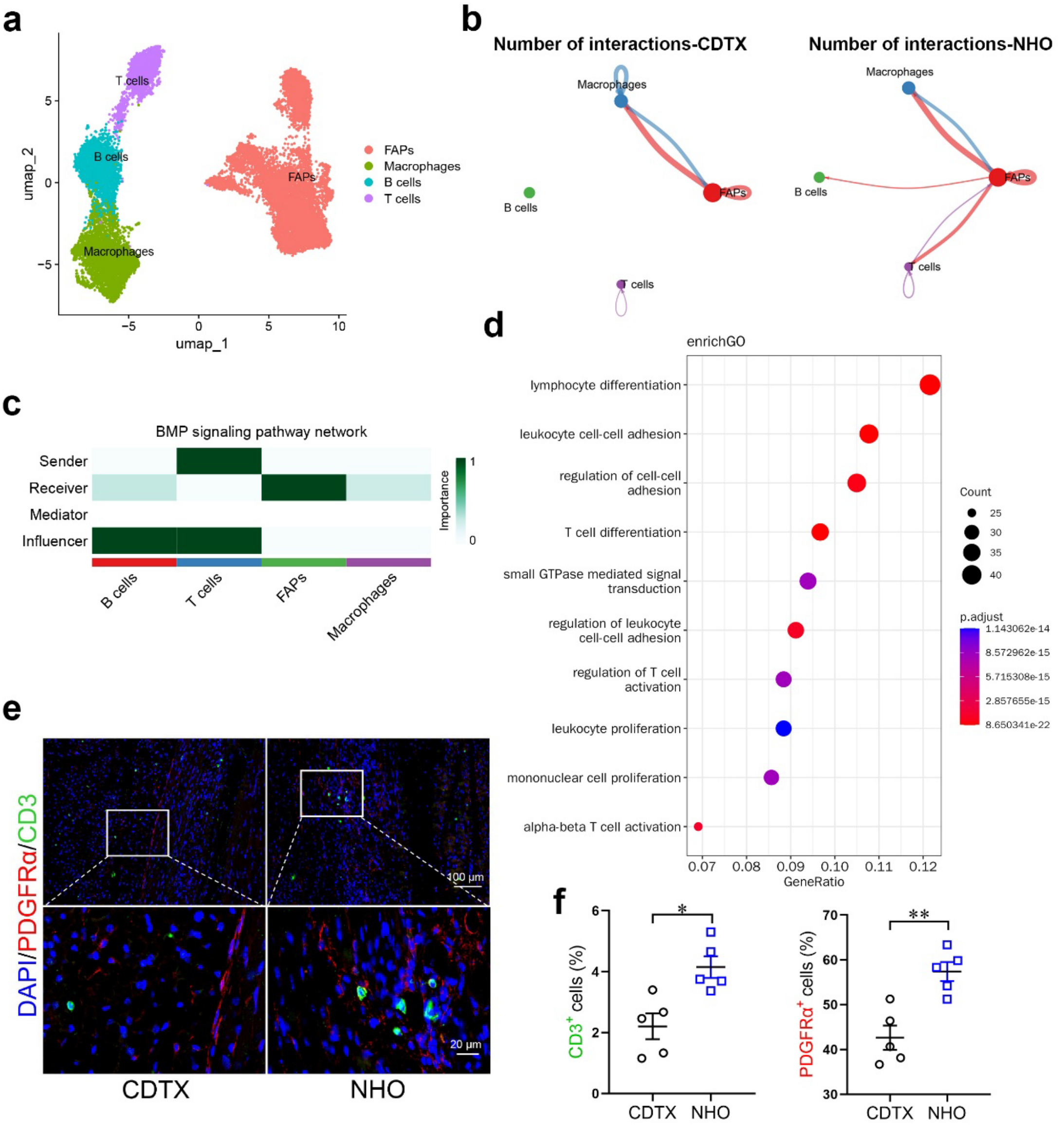

Supplementary Figure 3. The inter-cellular communication between T cells and FAPs.

a. The UMAP showed the FAPs, T cells, B cells, and macrophages of NHO and CDTX samples. (n=3)

b. The Circle plot showed the number of interactions among FAPs, T cells, B cells, and macrophages in NHO and CDTX samples.

c. Heatmap showed the interactions in BMP signaling pathways between B cells, T cells macrophages and FAPs in NHO samples.

d. The bubble chart showed Gene ontology (GO) analysis of DEGs in T cells.

e. IF staining of PDGFRα^{+} (red) cells and CD3^{+} (green) cells in the NHO and CDTX samples. Scale bars: 100μm (upper), 20μm (lower).

f. Quantification of the proportion of PDGFRα^+ cells and $CD3^+$ cells in E. $n = 5$, unpaired t-test. (The data were presented as the means ± SD. **$p < 0.01$ compared between groups; *$p < 0.05$ compared between groups).

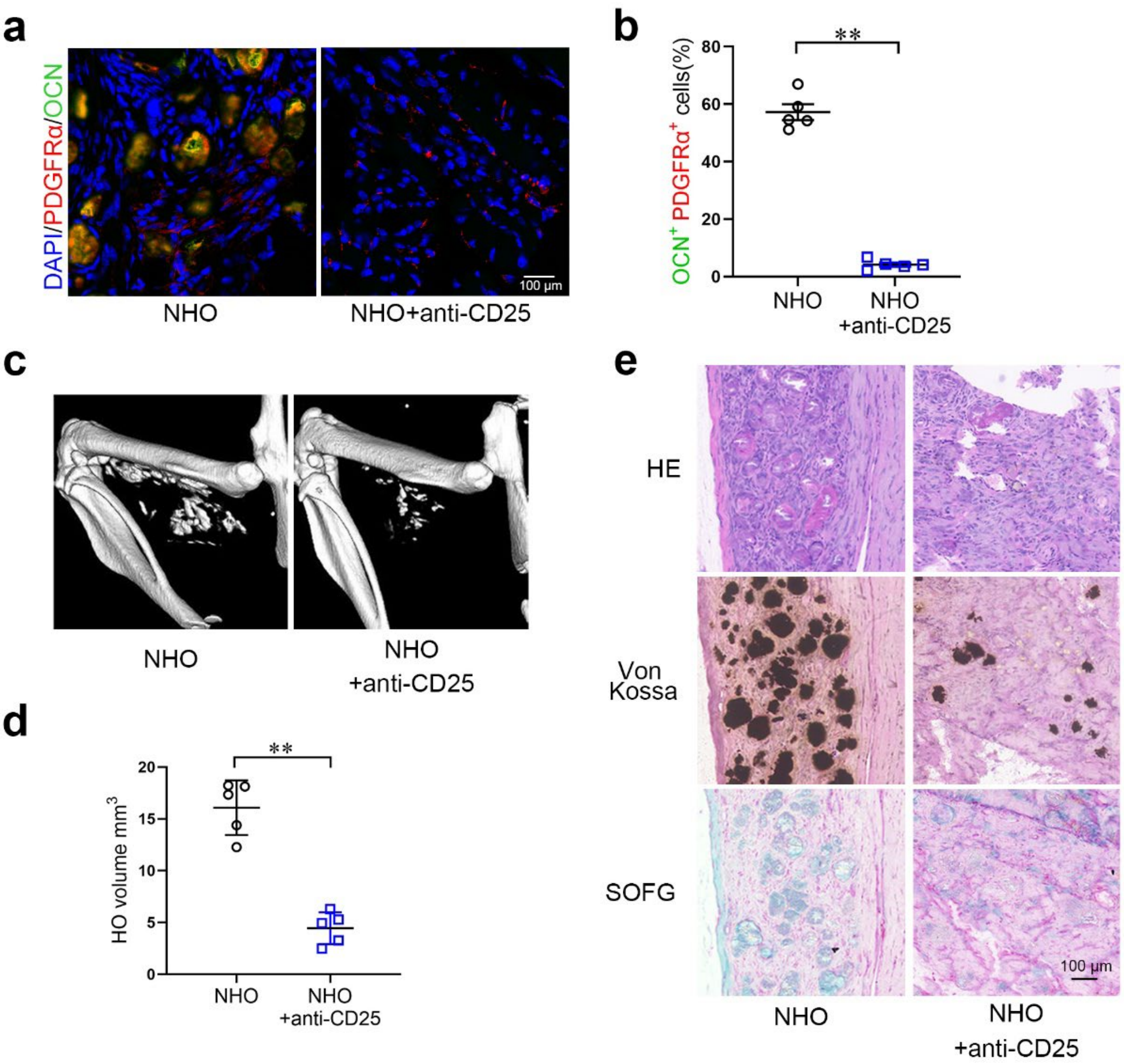

Supplementary Figure 4. Treg depletion significantly impedes ossification of FAPs in the injured muscle after SCI.

a.IF staining showed PDGFRα$^+$ (red) OCN$^+$ (green) cells in the NHO group and NHO + anti-CD25 group. Scale bars: 100μm.

b. Quantification of the proportion of PDGFRα$^+$OCN$^+$cells in A. n=5, unpaired t-test.

c. Micro-CT showed NHO of hindlimb muscle in NHO group and NHO + anti-CD25 group.

d. Quantitative analysis of NHO volumes by micro-CT analysis in C. n = 5, unpaired t-test.

e. H&E, Von Kossa and SOFG staining showed NHO of hindlimb muscle in NHO group and NHO + anti-CD25 group. Scale bars: 100μm. (The data were presented as the means ± SD. **p < 0.01 compared between groups).

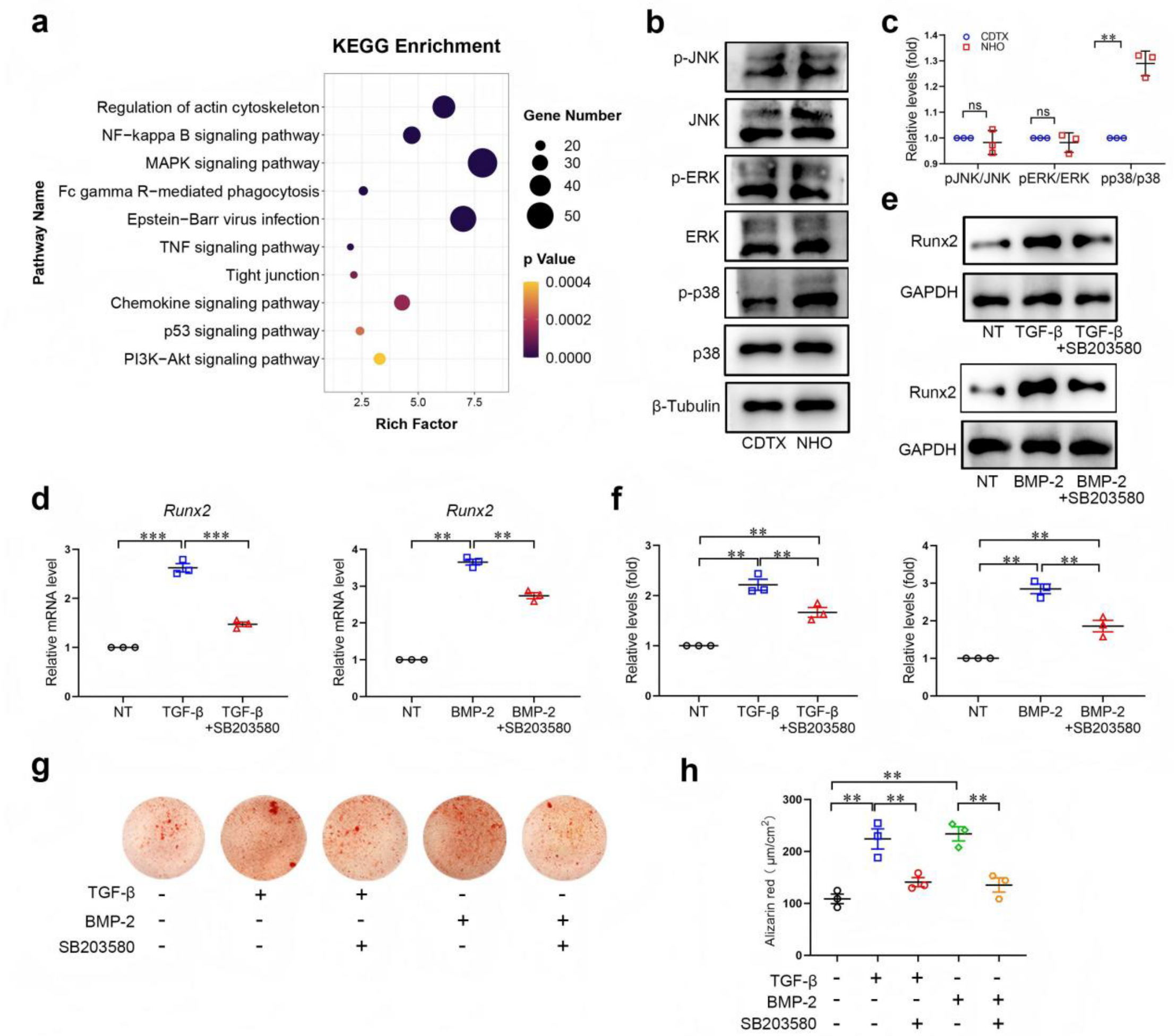

Supplementary Figure 5. Muscle-resident Treg-derived osteogenic factors activate MAPK/p38 signaling in FAPs to promote osteogenic differentiation.

a. KEGG enrichment analysis of differentially expressed genes in FAPs.

b. Western blot analysis of JNK, ERK and p38 MAPK phosphorylation in FAPs from CDTX and NHO groups. β-Tubulin was used as a loading control.

c. Quantification of p-JNK/JNK, p-ERK/ERK and p-p38/p38 protein levels in b. n = 3, unpaired t-test between CDTX and NHO groups for each protein ratio.

d. RT-qPCR analysis of *Runx2* mRNA expression in FAPs stimulated with TGF-β or BMP-2 in the presence or absence of SB203580. n = 3, one-way ANOVA, Bonferroni post hoc.

e. Western blot analysis of Runx2 protein expression in FAPs stimulated with TGF-β or BMP-2 in the presence or absence of SB203580. GAPDH was used as a loading control.

f. Quantification of RUNX2 protein levels in e. n = 3, one-way ANOVA with Bonferroni post hoc test.

g. Representative Alizarin Red S staining and quantification of calcium mineralization in FAPs treated with TGF-β or BMP-2 in the presence or absence of SB203580.

h. Quantification of calcium mineralization in e. $n = 3$, one-way ANOVA, Bonferroni post hoc. (Data are presented as mean ± SD. $^{**}P < 0.01$ compared between groups; $^{***}P < 0.001$ compared between groups.)

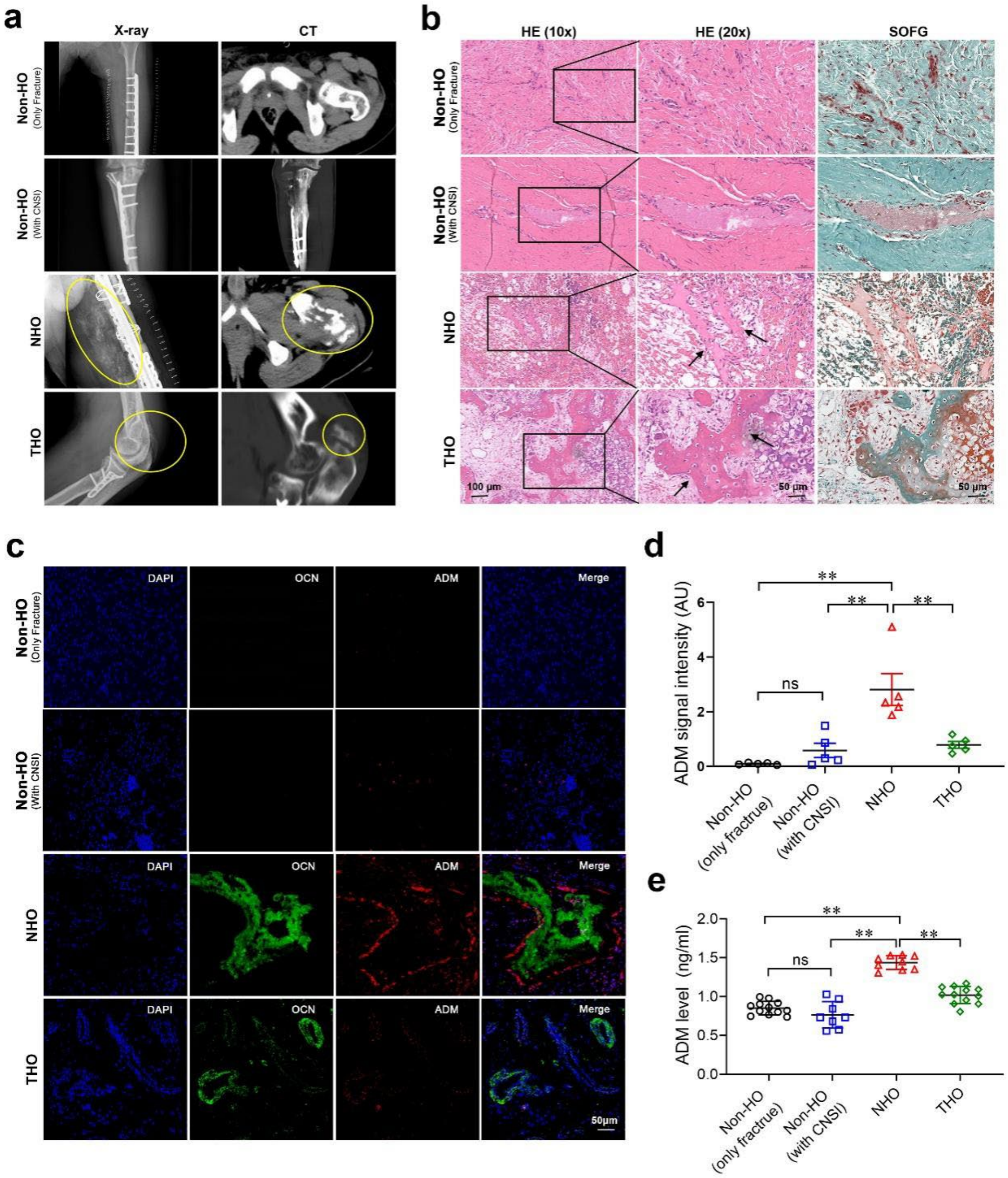

Supplementary Figure 6. ADM was increased in THO and NHO patients.

a. The X-ray and Micro-CT of NHO patients, Only SCI patients, THO patients and Only Fracture patients. The yellow boxes indicated HO formation.

b. H&E and SOFG staining of muscle samples from NHO patients, Only SCI patients, THO patients and Only Fracture patients. The black arrows indicated HO formation. Scale bars: 100μm (left), 50μm (middle and right).

c IF staining of OCN (green) and ADM (red) expression in muscle samples from NHO patients, Only SCI patients, THO patients and Only Fracture patients. Scale bars: 50μm.

d. Quantitative analysis of ADM signal intensity in C. $n = 5$, one-way ANOVA, Bonferroni post hoc.

e. The ELISA assays showed the ADM concentration in peripheral blood serum of NHO patients, Only SCI patients, THO patients and Only Fracture patients. (The data were presented as the means ± SD. $^{**}p < 0.01$ compared between groups).

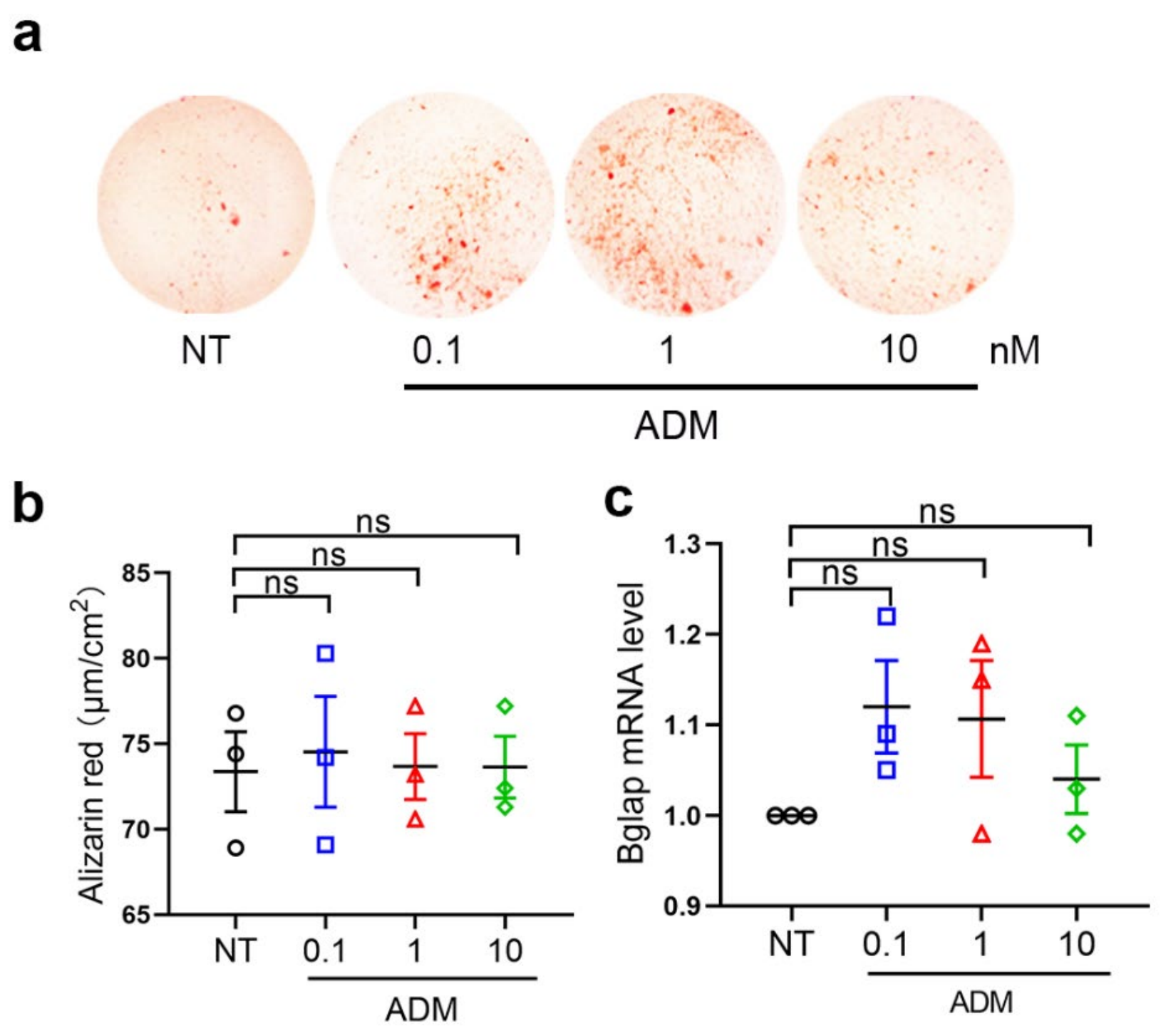

Supplementary Figure 7. ADM alone failed to induce osteogenic differentiation of FAPs *In vitro* experiments.

a. Alizarin Red S Staining showed Dose-response of ADM stimulation (0.1, 1 and 10nM) on calcium mineralization of FAPs in osteogenic medium.

b. Quantification of calcium mineralization expressed as the mean ± SD. n = 5, one-way ANOVA, Bonferroni post hoc.

c. RT-PCR analysis of mRNA expressions of osteogenesis marker *Bglap* in FAPs after stimulation with increasing concentrations of ADM for 24h. n = 3, one-way ANOVA, Bonferroni post hoc. (The data were presented as the means ± SD. "ns" represented "not significant", with a p-value > 0.05.).

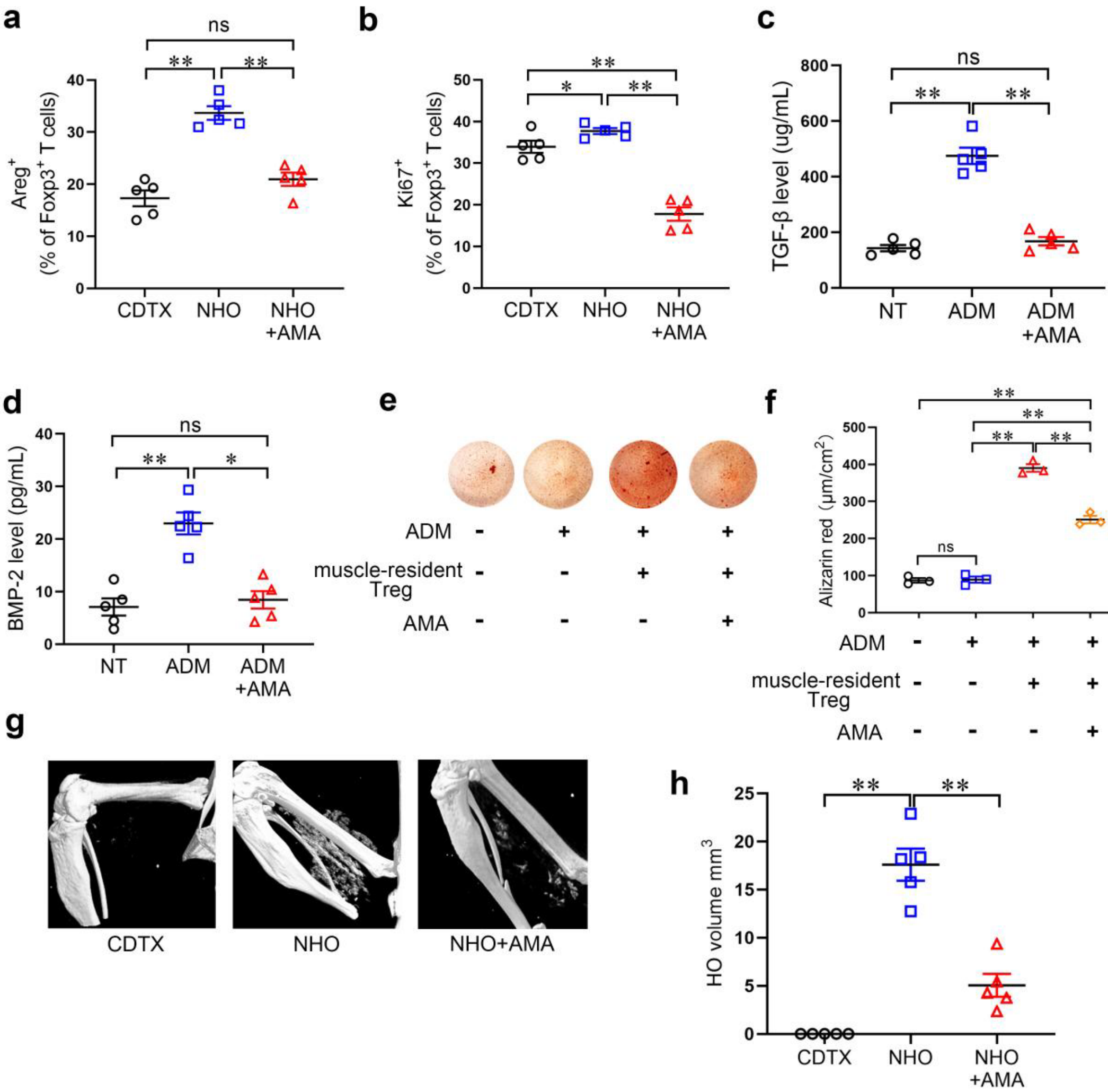

Supplementary Figure 8. Pharmacological blockade of ADM/RAMP2 signaling attenuates the pro-osteogenic function of muscle-resident Tregs.

a-b. Summary graph for the ex vivo characterization of muscle-residing Tregs in CDTX, NHO and NHO + AMA groups. (a) $Areg^+$ $Foxp3^+$ Treg;(b) $Ki67^+$ $Foxp3^+$ Treg. n = 5, one-way ANOVA, Bonferroni post hoc.

c-d. The ELISA assay showed the levels of TGF-β and BMP-2 in CM from muscle-resident Tregs treated with vehicle, ADM, or ADM plus AMA. n = 5, one-way ANOVA, Bonferroni post hoc.

e. Representative Alizarin Red S staining of FAPs under the indicated conditions. FAPs were treated with ADM alone or with CM from muscle-resident Tregs stimulated with ADM in the presence or absence of AMA.

f. Quantification of calcium mineralization in e. n = 5, one-way ANOVA, Bonferroni

post hoc.

g. Representative micro-CT images showing HO formation in the hindlimbs of CDTX, NHO and NHO + AMA mice.

h. Quantitative analysis of HO volume based on micro-CT images in g. $n = 5$, one-way ANOVA, Bonferroni post hoc. (The data were presented as the means ± SD. **$p < 0.01$ compared between groups; *$p < 0.05$ compared between groups; "ns" represented "not significant", with a p-value > 0.05.)

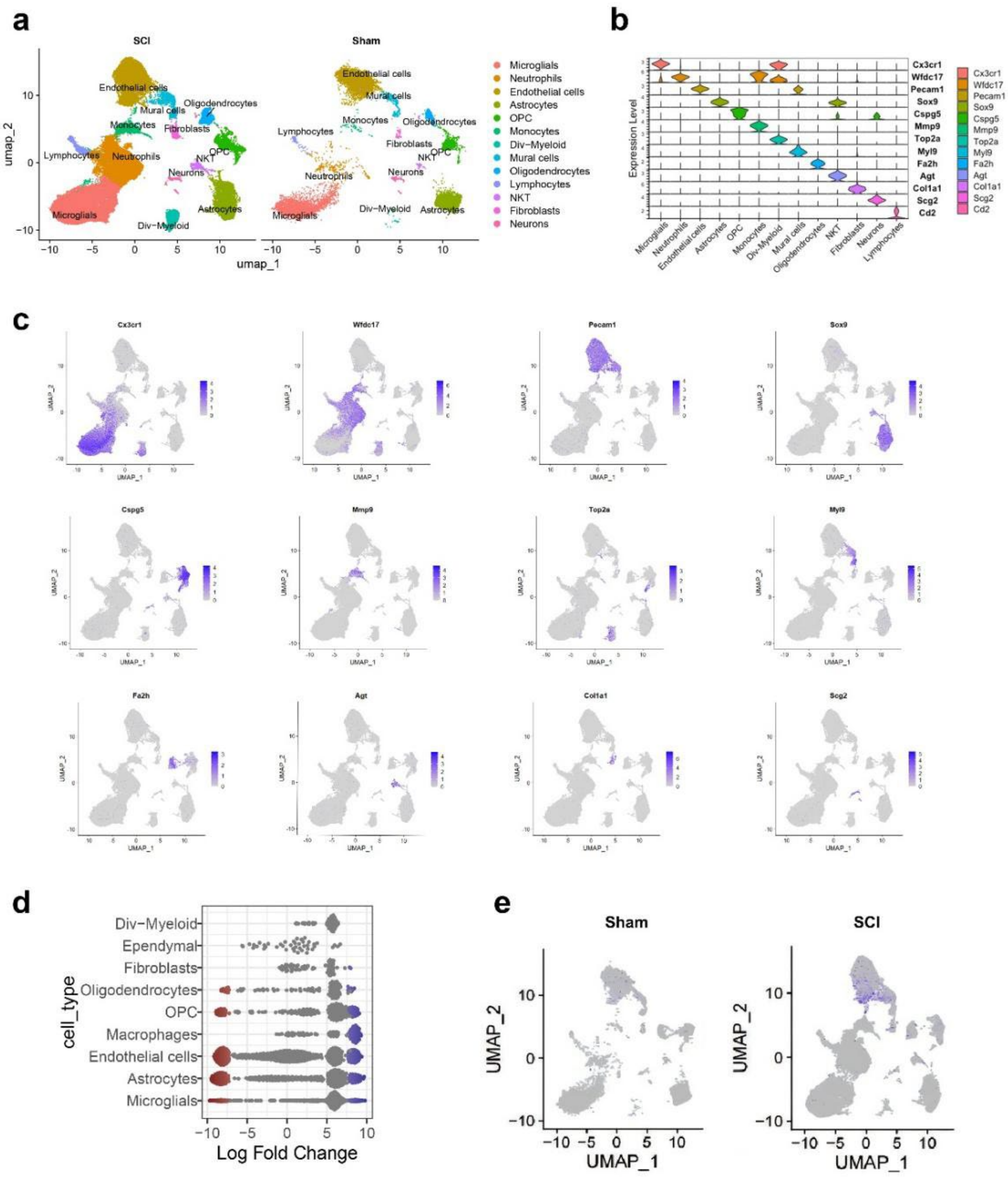

Supplementary Figure 9. ScRNA-seq showed the profile of spinal cord of SCI and sham groups.

a. The UMAP plot identified 13 main clusters of spinal cord tissue from SCI and sham groups.

b-c. The violin plot and UMAP plot showed the expression level of markers for 13 main clusters.

d. Milo analysis revealed differential abundance of cell neighborhoods in SCI versus sham groups.

e. The UMAP plot showed the expression of ADM in each cluster of SCI and sham groups.

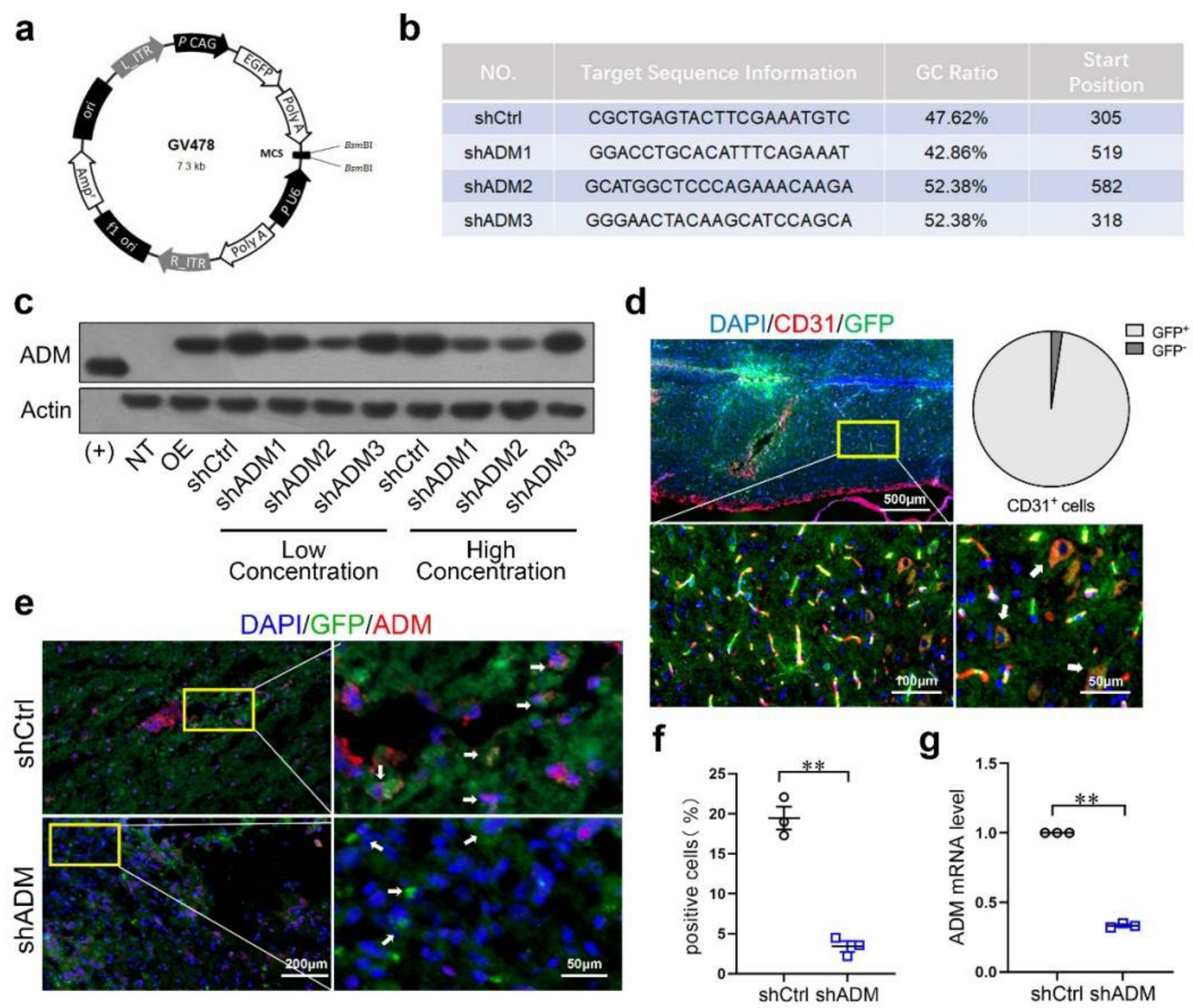

Supplementary Figure 10. Validation of infection efficiency of AAV-shADM.

a. Schematic representation of ADM knockdown using Adeno-associated virus (AAV).

b. The table shows the target sequence information, GC ratio and start position information for different groups of shCtrl, shADM1, shADM2 and shADM3.

c. Western blotting for ADM Protein levels in spinal cord of mice transfected with ADM shRNA in different sequences and concentration.

d. Horizontal sections of T9-T11 spinal segments. GFP signal was used to visualize AAV-mediated delivery of ADM co-expressing GFP (green). IF image and sector graph represent colocalization of GFP (green) and CD31 (red) in many cells (White arrows). Scale bars: 500μm (upper), 100μm (lower left), 20μm (lower right).

e. Representative images of ADM (red) and GFP (green) immunoreactivity in injured spinal cord in mice from AAV-shCtrl control group and AAV-shADM group. Scale bars: 100μm (left), 20μm (right).

f. Quantitative analysis of ADM^+ cells percentage in E. n = 3, unpaired t-test.

g. RT-PCR analysis of mRNA expressions of ADM in AAV-shCtrl control group and AAV-shADM group. n = 3, unpaired t-test. (The data were presented as the means ± SD. $^{**}p < 0.01$ compared between groups).

Table S1. Primers for RT-qPCR analysis of gene expression

Gene	Forward 5′-3′	Reverse 3′-5′
Pecam1	ACCGGGTGCTGTTCTATAAGG	TCACCTCGTACTCAATCGTGG
Apln	TGGCCCGGAATAGTCCCTAA	GGTGAGAGATGAGACCCCCT
Trp53i11	AGCCAGACGGATCTTGTGAG	TCCCGCACAGCAAACTTTATC
Adm	CACCCTGATGTTATTGGGTTCA	CCACTTATTCCACTTCTTTCGGA
Bglap	TGAGTCTGACAAAGCCTTC	CTGCTGTGACATCCATACTTG
Runx2	GACTGTGGTTACCGTCATGGC	ACTTGGTTTTTCATAACAGCGGA
Gapdh	AGGTCGGTGTGAACGGATTTG	TGTAGACCATGTAGTTGAGGTCA

Table S2. Reagent or resource

REAGENT or RESOURCE	SOURCE	IDENTIFIER
Antibodies		
PDGFRα	Abcam	Cat# ab32570
OCN	Abcam	Cat# ab93876
CD3	Abcam	Cat# ab135372
CD4-FITC	Biolegend	Cat# 116003
CD25-PE	Biolegend	Cat# 102007
Foxp3-BV421	Biolegend	Cat# 126419
Areg	Bioss	Cat# bs3847r
ST2	Abcam	Cat# ab25877
Ki67	Abcam	Cat# ab16667
Adrenomedullin	Bioss	Cat# bs-0007R
UCN2	ABclonal	Cat# A6485
RAMP2	Santa Cruz Biotechnology	Cat# sc-8853
PDGFRα-BB700	BD Biosciences	Cat# 742176
GR1- Biotin	Biolegend	Cat# 108404
CD3-Biotin	Biolegend	Cat# 100304
CD31-BV421	Biolegend	Cat# 102423
CD45-BV785	Biolegend	Cat# 103149
CD34-e660	eBioscience	Cat# 50-0341
Sca1-PECy7	Biolegend	Cat# 108114
ITGA7	Miltenyi Biotec	Cat# 130-103-357
APLN	Origene	Cat# TA351983S
RPS15	Abcam	Cat# ab249301
RPL29	Affinity Biosciences	Cat# DF9130
JNK	Cell Signaling Technology	Cat# #67096
pJNK	Cell Signaling Technology	Cat# #9251
ERK	Cell Signaling Technology	Cat# #4695
pERK	Cell Signaling Technology	Cat# #4370
p38	Cell Signaling Technology	Cat# #9212
p-p38	Cell Signaling Technology	Cat# #9211
Runx2	Cell Signaling Technology	Cat# ab192256
Tubulin	Cell Signaling Technology	Cat# ab6046
GAPDH	Cell Signaling Technology	Cat# ab181602
Bacterial and virus strains		
AAV9-shADM	GeneChem	
Chemicals, peptides, and recombinant proteins		
CDTX	LATOXAN	Cat# L8102
Alizarin red	Sigma-Aldrich	Cat# A5533
Adrenomedullin 22-52 (AMA)	MedChemExpress	Cat# HY-P1471
Recombinant human ADM	Sino Biological	Cat# 11646-H01H
Recombinant mouse VEGF	PeproTech	Cat# 100-20-10

Recombinant mouse TNFα	Sino Biological	Cat# 50349-MNAE
Recombinant mouse IL-1	Sino Biological	Cat# 50101-MNAE
Critical commercial assays		
Mouse ADM ELISA Kit	MEIMIAN	Cat# MM-0230M1
Mouse BMP-2 ELISA Kit	MEIMIAN	Cat# MM-0203M2
Mouse TGFβ ELISA Kit	MEIMIAN	Cat# MM_0689M1
Experimental models: Cell lines and Organisms/strains		
HUVECs	zqxzbio	Cat# DFSC-EC-01
C57BL/6J mice	Experimental Animal Center of Sun Yat-Sen University, Guangzhou, China	
RAPM2-flox mice	GemPharmatech	
Foxp3-Cre mice	GemPharmatech	
Oligonucleotides		
RT-qPCR primers	RibBio	

Table S3. List of RNAi sequences

Gene	Sequence
siRPL29	ACATGGCCAAGTCCAAGAAC
	TTAGGCCCTTTTTGTTGTGC
siRPS15	AAGGATCCAATGGCAGAAGTAGAGCAGA
	CCGAATTCTTACTTGAGAGGGATGAAG
shADM	ACCGGGCATGGCTCCCAGAAACAAGATTCAAGAGATCTTGTTTCTGGGAGCCATGCTTTTT
	TCTAAAAAAGCATGGCTCCCAGAAACAAGATCTCTTGAATCTTGTTTCTGGGAGCCATGCC

TableS4. Clinical characteristics of human subjects and correlation analysis between serum ADM levels and HO volume

Variable	Non-HO (only fractrue) (n=12)	Non-HO (with CNSI) (n=8)	NHO (n=9)	THO (n=12)	*P* value
**Age, years** [a]	40.17±6.56	38.00±7.93	41.56±6.19	38.67±5.79	0.6597, ns
**Sex** [b]					0.5661, ns
Male	8	3	6	7	
Female	4	5	3	5	
**Injruy Time, days** [a]	26.67±4.23	25.88±4.91	28.11±4.37	26.92±3.83	0.7509, ns
**NSAIDs** [b]					0.2520, ns
Yes	6	4	7	4	
No	6	4	2	8	
**ADM ng/ml** [a]	0.85±0.085	0.77±0.17	1.44±0.09	1.02±0.11	< 0.0001, ****
**HO volume cm^3**	0	0	27.70±7.72	18.89±6.50	< 0.0001, ****
**Correlation** [c]					
r			0.8817	0.5618	
P value			0.0017, **	0.0573, ns	
95% confidence interval			0.5249 to 0.9749	-0.01792 to 0.8588	

ADM, adrenomedullin; HO, heterotopic ossification; NHO, neurological heterotopic ossification; THO, traumatic heterotopic ossification; CT, computed tomography; NSAIDs, non-steroidal anti-inflammatory drugs; r, Pearson correlation coefficient; SD, standard deviation; CI, confidence interval.

The data were presented as the mean ± SD. **$p < 0.01$; ****$p < 0.0001$; "ns" represented "not significant", with a p-value > 0.05. [a] One-way ANOVA test, [b] Fisher's precision test, [c] Pearson test.
